# Fragment binding to the Nsp3 macrodomain of SARS-CoV-2 identified through crystallographic screening and computational docking

**DOI:** 10.1126/sciadv.abf8711

**Published:** 2021-04-14

**Authors:** Marion Schuller, Galen J. Correy, Stefan Gahbauer, Daren Fearon, Taiasean Wu, Roberto Efraín Díaz, Iris D. Young, Luan Carvalho Martins, Dominique H. Smith, Ursula Schulze-Gahmen, Tristan W. Owens, Ishan Deshpande, Gregory E. Merz, Aye C. Thwin, Justin T. Biel, Jessica K. Peters, Michelle Moritz, Nadia Herrera, Huong T. Kratochvil, QCRG Structural Biology Consortium, Anthony Aimon, James M. Bennett, Jose Brandao Neto, Aina E. Cohen, Alexandre Dias, Alice Douangamath, Louise Dunnett, Oleg Fedorov, Matteo P. Ferla, Martin R. Fuchs, Tyler J. Gorrie-Stone, James M. Holton, Michael G. Johnson, Tobias Krojer, George Meigs, Ailsa J. Powell, Johannes Gregor Matthias Rack, Victor L. Rangel, Silvia Russi, Rachael E. Skyner, Clyde A. Smith, Alexei S. Soares, Jennifer L. Wierman, Kang Zhu, Peter O’Brien, Natalia Jura, Alan Ashworth, John J. Irwin, Michael C. Thompson, Jason E. Gestwicki, Frank von Delft, Brian K. Shoichet, James S. Fraser, Ivan Ahel

**Affiliations:** 1 Sir William Dunn School of Pathology, University of Oxford, South Parks Road, Oxford OX1 3RE, UK.; 2 Department of Bioengineering and Therapeutic Sciences, University of California San Francisco, San Francisco, CA 94158, USA.; 3 Department of Pharmaceutical Chemistry, University of California San Francisco, San Francisco, CA 94158, USA.; 4 Diamond Light Source Ltd., Harwell Science and Innovation Campus, Didcot OX11 0DE, UK.; 5 Institute for Neurodegenerative Disease, University of California San Francisco, San Francisco, CA 94158, USA.; 6 Chemistry and Chemical Biology Graduate Program, University of California San Francisco, San Francisco, CA 94158, USA.; 7 Tetrad Graduate Program, University of California San Francisco, San Francisco, CA 94158, USA.; 8 Quantitative Biosciences Institute (QBI) Coronavirus Research Group Structural Biology Consortium, University of California San Francisco, San Francisco, CA 94158, USA.; 9 Biochemistry Department, Institute for Biological Sciences, Federal University of Minas Gerais, Belo Horizonte, Brazil.; 10 Helen Diller Family Comprehensive Cancer, University of California San Francisco, San Francisco, CA 94158, USA.; 11 Centre for Medicines Discovery, University of Oxford, South Parks Road, Headington OX3 7DQ, UK.; 12 Stanford Synchrotron Radiation Lightsource, SLAC National Accelerator Center, Menlo Park, CA 94025, USA.; 13 Wellcome Centre for Human Genetics, University of Oxford, Old Road Campus, Oxford OX3 7BN, UK.; 14 National Synchrotron Light Source II, Brookhaven National Laboratory, Upton, NY 11973, USA.; 15 Department of Biochemistry and Biophysics, University of California San Francisco, San Francisco, CA 94158, USA.; 16 Department of Molecular Biophysics and Integrated Bioimaging, Lawrence Berkeley National Laboratory, Berkeley, CA 94720, USA.; 17 ChemPartner Corporation, South San Francisco, CA 94080, USA.; 18 Structural Genomics Consortium, University of Oxford, Old Road Campus, Roosevelt Drive, Headington OX3 7DQ, UK.; 19 School of Pharmaceutical Sciences of Ribeirao Preto, University of Sao Paulo, São Paulo, Brazil.; 20 Photon Sciences, Brookhaven National Laboratory, Upton, NY 11973, USA.; 21 Department of Chemistry, University of York, Heslington, York YO10 5DD, UK.; 22 Department of Cellular and Molecular Pharmacology, University of California San Francisco, San Francisco, CA 94158, USA.; 23 Department of Chemistry and Biochemistry, University of California Merced, Merced, CA 95343, USA.; 24 Department of Biochemistry, University of Johannesburg, Auckland Park 2006, South Africa.

## Abstract

The severe acute respiratory syndrome coronavirus 2 (SARS-CoV-2) macrodomain within the nonstructural protein 3 counteracts host-mediated antiviral adenosine diphosphate–ribosylation signaling. This enzyme is a promising antiviral target because catalytic mutations render viruses nonpathogenic. Here, we report a massive crystallographic screening and computational docking effort, identifying new chemical matter primarily targeting the active site of the macrodomain. Crystallographic screening of 2533 diverse fragments resulted in 214 unique macrodomain-binders. An additional 60 molecules were selected from docking more than 20 million fragments, of which 20 were crystallographically confirmed. X-ray data collection to ultra-high resolution and at physiological temperature enabled assessment of the conformational heterogeneity around the active site. Several fragment hits were confirmed by solution binding using three biophysical techniques (differential scanning fluorimetry, homogeneous time-resolved fluorescence, and isothermal titration calorimetry). The 234 fragment structures explore a wide range of chemotypes and provide starting points for development of potent SARS-CoV-2 macrodomain inhibitors.

## Teaser

Massive fragment screening effort provides a foundation for the development of macrodomain inhibitors as novel antiviral agents.

## INTRODUCTION

Macrodomains are conserved protein domains found in all kingdoms of life and in several viruses (*1*). Viral macrodomains recognize and remove host-derived adenosine diphosphate (ADP)–ribosylation, a posttranslational modification of host and pathogen proteins (*2*, *3*). The innate immune response involves signaling by ADP-ribosylation, which contributes to the suppression of viral replication (*3*–*7*). Upon viral infection, ADP-ribosylation is catalyzed by an interferon-induced subset of mammalian ADP-ribosyltransferases, collectively termed “antiviral poly(ADP-ribosyl) polymerases” (PARPs) (*3*, *8*). These enzymes transfer the ADP-ribose (“ADPr”) moiety of nicotinamide adenine dinucleotide onto target proteins (*3*, *8*). For example, during coronavirus (CoV) infection, PARP14 stimulates interleukin-4–dependent transcription, which leads to the production of proinflammatory, antiviral cytokines (*9*). Viral macrodomains, which are found primarily in corona, alpha, rubi, and herpes viruses, can counteract this host defense mechanism via their (ADP-ribosyl)hydrolase activity, contributing to the host-viral arms race for control of cell signaling (*10*).

CoVs are important pathogens of livestock and humans. Three strains of seven known to infect humans have caused major outbreaks within the past two decades: the severe acute respiratory syndrome (SARS) CoV, causing the SARS epidemic from 2002 to 2004; the Middle East respiratory syndrome CoV, causing outbreaks in 2012, 2015, and 2018; and SARS-CoV-2, causing the current CoV disease 2019 (COVID-19) pandemic (*11*). The coronaviral conserved macrodomain (called “Mac1” here; also known as “S2-MacroD” or “X domain”) is encoded as part of the nonstructural protein 3 (Nsp3), a 200-kDa multidomain protein (*12*). While cell culture experiments suggest that SARS Mac1 is dispensable for viral replication in some cell lines (*5*, *13*, *14*), animal studies have shown that its hydrolytic activity promotes immune evasion and that it is essential for viral replication and pathogenicity in the host (*6*, *7*). The critical role of macrodomains is further supported by experiments using catalytic null mutations of the murine hepatitis virus, which render that virus essentially nonpathogenic (*5*, *6*, *13*). Collectively, these findings support the idea that SARS-CoV-2 Mac1 is a promising drug target for disrupting the viral life cycle.

A barrier for macrodomain drug discovery has been the lack of well-behaved inhibitors for this domain. Making matters worse, there are few biochemical assays suitable for screening for these inhibitors. Thus far, PDD00017273, an inhibitor of the poly(ADPr)glycohydrolase (PARG), a macrodomain-type (ADP-ribosyl)hydrolase, remains the only well-characterized inhibitor with convincing on-target pharmacology and selectivity (*15*). The initial hit was found by a homogeneous time-resolved fluorescence (HTRF)–based assay that measures PARG activity, rendering the assay unsuitable for macrodomains that lack this activity (*16*). A selective allosteric inhibitor targeting PARP14 was identified in an AlphaScreen-based high-throughput screen (HTS) (*17*). While this inhibitor showed on-target activity in cells, its unique allosteric binding site is difficult to translate to other macrodomains. While potential Mac1 inhibitors have emerged with the advent of SARS-CoV-2 (*18*), their binding mechanisms and efficacy remain unclear, and the lack of a biochemical assay specific for Mac1 has hindered their development. Furthermore, structures of the new inhibitors bound to Mac1 have not yet been reported, making optimization of initial hits, however promising, difficult.

**Fig. 1:**
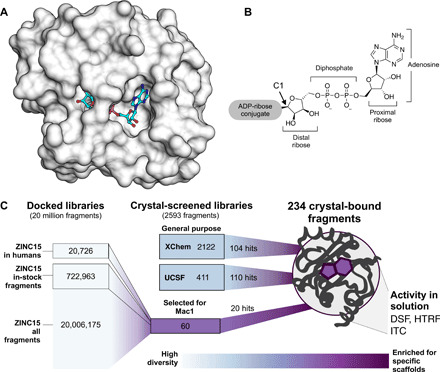
Overview of the fragment discovery approach for SARS-CoV-2 Nsp3 Mac1 presented in this study. (**A**) Surface representation of Nsp3 Mac1 with ADPr bound (cyan) in a deep and open binding cleft. (**B**) Nsp3 Mac1 has (ADP-ribosyl)hydrolase activity, which removes ADP-ribosylation modifications attached to host and pathogen targets. ADPr is conjugated through C1 of the distal ribose. (**C**) Summary of the fragment discovery campaign presented in this work. Three fragment libraries were screened by crystallography: two general-purpose [XChem and University of California San Francisco (UCSF)] and a third bespoke library of 60 compounds, curated for Mac1 by molecular docking of more than 20 million fragments. Crystallographic studies identified 214 unique fragments binding to Mac1, while the molecular docking effort yielded 20 crystallographically confirmed hits. Several crystallographic and docking fragments were validated by isothermal titration calorimetry (ITC), differential scanning fluorimetry (DSF), and a HTRF-based ADPr-peptide displacement assay.

To address the lack of chemical matter against Mac1, we turned to fragment-based ligand discovery using crystallography as a primary readout (Fig. 1). Fragment screens can efficiently address a large and relatively unbiased chemical space (*19*). Despite typically weak overall affinity, fragments often have high ligand efficiency (LE) [−Δ*G*_b_/HAC (heavy atom count)] and can provide templates for further chemical elaboration into lead-like molecules (*20*). Crystallography can be used as a primary screening method for fragment discovery (*21*), and recent automation and processing software at synchrotron radiation sources has made this routinely possible at facilities such as the XChem platform at Diamond Light Source (*22*–*25*). As part of Diamond Light Source’s contribution toward efforts to combat COVID-19, fragment screening expertise and infrastructure were made immediately available to any users working on SARS-CoV-2 targets (*26*). Similarly, synchrotron access for essential COVID-19–related research was also made available at the U.S. Department of Energy light sources.

Because crystallographic fragment screens can generate hits that bind anywhere on the protein surface, we wanted to supplement those screens with molecular docking intentionally targeting the active site. Docking has the additional benefit of exploring a much larger chemical space than an empirical fragment library. While an empirical library of ~1000 to 2000 fragments can represent a chemical space as large as, or larger than, that of a classic HTS library of several million compounds, exploration of chemotypes, including those that are well suited to a particular target subsite, will inevitably be limited (*27*). Conversely, docking a much larger virtual library allows finer-grained sampling around many chemotypes. A potential drawback of molecular docking is doubt about its ability to predict weakly binding fragment geometries with high fidelity. While docking has identified potent ligands from libraries of lead-like molecules [250 to 350 atomic mass units (amu)] (*28*–*30*), these molecules offer more functional group handles for protein matching than do most fragments (150 to 250 amu), and docking is thought to struggle with the smaller, less complex, and geometrically more promiscuous fragments (*31*). Thus, the pragmatism of this approach has been uncertain (*32*, *33*).

Here, we present a combination of experimental crystallographic-based and computational docking–based fragment screens performed against Nsp3 Mac1 of SARS-CoV-2 (Fig. 1). Using x-ray crystallography, we screened fragment libraries of 2533 compounds, yielding 214 unique fragment-bound Mac1 structures at atomic resolution. Docking of more than 20 million compounds prioritized 60 molecules for structure determination, yielding the structures of 20 additional compounds bound to Mac1. Additional x-ray data collection to ultrahigh resolution and at physiological temperature illuminated the conformational heterogeneity in the Mac1 active site. We were able to confirm the binding of several fragments with differential scanning fluorimetry (DSF), isothermal titration calorimetry (ITC), and an HTRF-based ADPr-peptide displacement assay, validating the activity of these molecules and providing a foundation for their optimization. The new fragments explore a wide range of chemotypes that interact with the catalytic site of Mac1. Together, these results create a road map for inhibitor development against Mac1, which may help to combat the pathogenicity of SARS-CoV-2.

## RESULTS

### Two crystal forms of Nsp3 Mac1 reveal differences in active site accessibility

We sought a crystal system that enabled consistent ligand soaking for fragment screening and for testing docking predictions. Six Mac1 crystal forms have previously been reported (data file S1). Initially, we designed a construct based on Protein Data Bank (PDB) entry 6VXS (*34*). This construct has been reported to crystallize in P1, C2, and P2_1_ with either one or two molecules in the asymmetric unit (ASU) (data file S1). This construct crystallized reproducibly in C2 with microseeding and diffracted to a maximum resolution of 0.77 Å (data file S1 and figs. S1 and S2A). This high-resolution data yielded electron density maps at true atomic resolution with abundant alternative conformations (fig. S1). The electron density maps also revealed features that are rarely observed in macromolecular crystallography, such as explicit hydrogen atoms, and covalent bond density (fig. S1). Although the active site appears accessible (fig. S3B), efforts to soak ADPr into the crystals were unsuccessful. In addition, soaking revealed that this crystal form suffers from inconsistent dimethyl sulfoxide (DMSO) tolerance (fig. S2A), which is problematic for fragment soaking. In attempts to overcome this problem, we experimented with lysine methylation (*35*), which increased DMSO tolerance (fig. S2A) but unfortunately increased occlusion of the active site (fig. S3, F and G), and dehydration, which increased DMSO tolerance, at the cost of nonisomorphism (fig. S2, A and C).

In parallel, we designed a new Mac1 construct that crystallized in P4_3_ with two molecules in the ASU (data file S1). This construct crystallized reproducibly with microseeding and diffracted to a maximum resolution of 0.85 Å (data file S1). The sequence differences between the two constructs were slight (data file S1) yet resulted in a substantially different crystal packing (fig. S3, B to E). Although the active site of protomer B was obstructed, the active site of protomer A was accessible (fig. S3B), and we were able to soak ADPr into the crystals (fig. S4A). This new structure also revealed a notable difference compared to previously reported Mac1-ADPr structures: The α-anomer of the terminal ribose was observed instead of the β-anomer (fig. S4, A to D). Despite this, alignment of ADPr is excellent between all Mac1-ADPr structures (fig. S4D), and the structures are similar overall (fig. S4E). The DMSO tolerance of the P4_3_ crystals was excellent (fig. S2A). Accordingly, most of our fragment soaking work proceeded with this construct.

### Identifying new ligands for Nsp3 Mac1 using crystallographic fragment screening and docking

#### Characterization of experimental and virtual screening libraries

Crystal soaking screens at the XChem facility were performed with the P4_3_ crystals and a collection of fragment libraries [e.g., Diamond Light Source, Structural Genomics Consortium (SGC), and iNEXT (DSI)–poised library including 687 molecules (*36*) and the EU-OPENSCREEN containing 968 molecules] totaling 2122 molecules (see data file S1 for details). Crystals were screened at the Diamond Light Source. At University of California San Francisco (UCSF), a fragment library composed of Enamine’s Essential Fragment library with 320 compounds, augmented by an additional 91 molecules from an in-house library (UCSF_91), was screened against both the P4_3_ and C2 crystal forms at the Advanced Light Source (ALS), the Stanford Synchrotron Radiation Lightsource (SSRL), and the National Synchrotron Light Source II (NSLS-II). On average, molecules across the XChem and UCSF collections had molecular weights of 192 ± 47 amu, cLog*P* values from −1.8 to 3.8, 13 ± 3 heavy atoms, and, on average, two rotatable bonds (fig. S5).

Two fragment libraries were computationally docked against the structure of Mac1 (PDB 6W02): a library of 722,963 fragments “in stock” at commercial vendors and the entire ZINC15 fragment library of 20,006,175 mainly make-on-demand fragments that have not been previously synthesized, but can readily be made, available predominantly from Enamine and WuXi (*34*). Molecules from the ZINC15 fragment library had molecular weights of <250 amu, cLog*P* < 3.5, with an average of four rotatable bonds, and typically 4 to 19 heavy atoms (fig. S5). In addition, an “in-human” library of 20,726 drugs, investigational new drugs, and metabolites that have been tested in humans were included into the docking screen, with a view to potential repurposing opportunities. All three sets can be downloaded from ZINC15 (http://zinc15.docking.org) (*37*).

We investigated the fragment libraries for their diversity and their representation of chemotypes likely to bind at the adenine recognition site of Mac1 (fig. S5). Bemis-Murcko (BM) scaffold (*38*) analysis revealed 179 unique scaffolds in the UCSF libraries and 809 such scaffolds in the XChem fragment libraries. The in-stock fragment docking library contained 69,244 scaffolds, while 803,333 scaffolds were present in the entire ZINC15 20M fragment collection. Together, the experimentally screened libraries contained roughly two compounds per BM scaffold, while the docking libraries contained approximately 10 fragments per scaffold, consistent with the expected higher granularity of the docking libraries afforded by their much larger size.

Because adenine-containing compounds are the only structurally characterized binders of Mac1 and fragment libraries are intended to cover a wide chemotype space, we assessed the prevalence of pyrimidines in the libraries. We found pyrimidines in 12 of the 411 fragments in the UCSF libraries and in 72 of the 2122 XChem fragments (3.39% of the physically screened fragments) (fig. S5). Pyrimidines were found in 41,531 of the 722,963 (5.74%) in-stock fragments and in 890,199 molecules of the 20,006,175 compound fragment library (4.44%). While the percentages of molecules carrying the pyrimidine substructure were similar between the physical and docked fragments, the absolute numbers in the latter sets were far higher. Aside from bearing a pyrimidine substructure, these subsets were otherwise diverse: Among the 890,199 pyrimidine-containing docking fragments, 60,919 distinct BM scaffolds were identified. Adenine itself was present in 5457 fragments (582 different scaffolds). Furthermore, as ADPr is negatively charged, anionic compounds were considered to exhibit favorable properties to bind to Mac1 by targeting the diphosphate region. Fortuitously, a substantial fraction (35%) of the UCSF fragment libraries is anionic (fig. S5).

#### Hit rates and Mac1 interaction sites of fragments

**Fig. 2:**
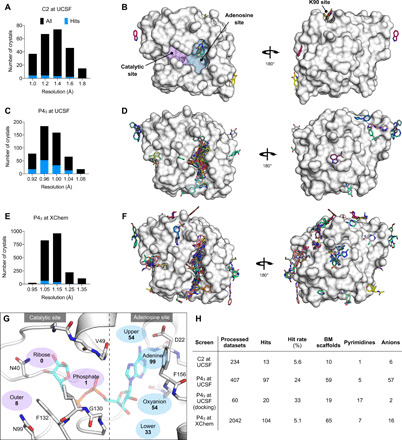
Crystallographic screening identified 234 fragments bound to Mac1. (**A**, **C**, and **E**) Histograms showing the resolution of the crystallographic fragment screening data. The resolution of datasets where fragments were identified are shown with blue bars. (**B**, **D**, and **F**) Surface representation of Mac1 with fragments shown as sticks. (**G**) The Mac1 active site can be divided on the basis of the interactions made with ADPr. The “catalytic” site recognizes the distal ribose and phosphate portion of the ADPr and harbors the catalytic residue Asn^40^ (*10*). The “adenosine” site recognizes adenine and the proximal ribose. The number of fragments binding in each site is indicated. (**H**) Summary of the fragments screened by x-ray crystallography, including the number of BM scaffolds and anionic fragments identified as hits in each screen. “Processed datasets” refers to the number of datasets that were analyzed for fragment binding with PanDDA. Of the datasets collected for 2954 fragments, 211 (7.1%) were not analyzed because of data pathologies.

Across both crystal forms and facilities, we collected diffraction data for Mac1 crystals soaked with 2954 fragments (data file S1). The diffraction characteristics of the P4_3_ crystals were excellent: The average resolution was 1.1 Å, and 98% of the crystals diffracted beyond 1.35 Å (Fig. 2, C and E, and fig. S2B). Although diffraction data were collected for 368 fragments soaked into the C2 crystals at UCSF, data pathologies meant that only 234 datasets could be analyzed. The datasets collected from C2 crystals had a mean resolution of 1.4 Å and ranged from 1.0 to 2.2 Å (Fig. 2A and fig. S2B). In total, we identified 234 unique fragments binding to Mac1 using the PanDDA (pan-dataset density analysis) method (Fig. 2 and data files S1 and S2) (*39*). Of these, 221 were identified using P4_3_ crystals (hit rate of 8.8%) and 13 using C2 crystals (hit rate of 5.6%). Eighty percent of the fragments were identified in the Mac1 active site, near to or overlapping with the regions occupied by the nucleoside (the adenosine site) or the phosphoribose (the catalytic site) (Fig. 2G). Additional fragments were scattered across the surface of the enzyme, with an enrichment at a distal macrodomain-conserved pocket near lysine 90 (the “K90 site,” 14 fragments) and with many others stabilized by crystal contacts (Fig. 2, B, D, and F, and fig. S6). Coordinates, structure factors, and PanDDA electron density maps for all the fragments have been deposited in the PDB and are available through the Fragalysis webtool (https://fragalysis.diamond.ac.uk).

The unusually high hit rate for the adenosine site in the P4_3_ form with the Enamine Essential library (21%) was in contrast to the relatively low hit rate with this library with the C2 form (1.3%). Of the five pairs of fragments identified in both crystal forms, two pairs were identified in the adenine subsite in both crystal forms, two in the adenine subsite in P4_3_ crystals but in the K90 site in C2 crystals, and the remaining pair bound to a surface site in the P4_3_ crystals and in the K90 site in the C2 crystals (data file S1). Additional paired high-quality datasets were available for 54 fragments that were bound within the P4_3_ crystals, but all showed no density for fragments in the C2 crystals (data file S1). It is possible that competition for binding with the N-terminal residues may have contributed to the relatively low hit rate for the C2 form (fig. S3F).

#### Docking hits mimic the adenine recognition pattern

Docking the entire (20 million) ZINC15 fragment library, after calibration of docking parameters using different control calculations (see Materials and Methods) (*37*, *40*), was completed in approximately 5 hours of elapsed time on 500 cores. The 20,006,175 fragments were sampled in over 4.4 trillion complexes. Top-ranked molecules were inspected for their ability to form hydrogen bonds similar to adenine (e.g., with the side chain of Asp^22^ and with the backbones of Ile^23^ and Phe^156^), while molecules with internal molecular strain or unsatisfied hydrogen bond donors were deprioritized. Ultimately, we selected 54 fragments from the entire ZINC15 fragment library screen, 9 of which were immediately available for purchase from Enamine and 33 of 45 make-on-demand molecules were successfully synthesized de novo. Furthermore, eight fragments were purchased from the ZINC15 in-stock fragment library screen, and an additional 10 compounds were sourced on the basis of the in-human library docking (data file S1).

**Fig. 3:**
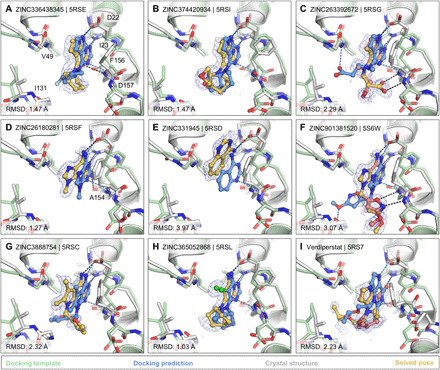
Docking hits confirmed by high-resolution crystal structures. The protein structure (PDB 6W02) (*34*) prepared for virtual screens is shown in green, predicted binding poses are shown in blue, the crystal protein structures are shown in gray, and the solved fragment poses are shown in yellow, with alternative conformations shown in light pink. PanDDA event maps are shown as a blue mesh. Event maps were calculated before ligand modeling, and the maps are free from model bias toward any ligand (*39*). Protein-ligand hydrogen bonds predicted by docking or observed in crystal structures are colored light blue or black, respectively. Hungarian RMSD values are presented between docked and crystallographically determined ligand poses (binding poses for additional docking hits are shown in fig. S7).

Of the 60 molecules tested for complex formation by crystal soaking, 20 were observed with unambiguous electron density in complex with Mac1 (data file S1). Here too, the crystals diffracted to exceptionally high resolution, between 0.94 and 1.01 Å. The predicted docking poses typically superposed well on the observed crystallographic results [Hungarian method root mean square deviations (RMSDs) (*41*) ranging from 1 to 5 Å] and 19 of the 20 docking hits bound to the adenine subsite of Mac1, as targeted by docking (Fig. 3 and fig. S7).

The most commonly observed scaffold among the docking hits was 7*H*-pyrrolo(2,3-*d*)pyrimidine occupying the adenine-binding subsite (Fig. 3, A to C, and fig. S7, A and B). This ring system is typically hydrogen bonded with Asp^22^, Ile^23^, and Phe^156^. Fragments with this scaffold usually demonstrated high fidelity between the docking results and the high-resolution structures (RMSD, 1.5 to 2.3 Å). For RMSD values of >2 Å, indicating noticeable deviations between docking and crystallography (*42*), visual inspection of docked and solved poses still revealed correct predictions of orientation and key interactions for most fragments in the targeted binding subsite (e.g., Fig. 3, C, F, and G). Different substituents can be attached to this headgroup, e.g., piperidine, adding a hydrophobic segment to the scaffold [e.g., ZINC336438345 (PDB 5RSE)], occupying most of the adenosine-binding site, as shown in Fig. 3 (A and B) and fig. S7 (A and B). In addition to hydrophobic variations, ZINC263392672 (PDB 5RSG) attaches an anionic substituent to the pyrrolopyrimidine scaffold, offering additional hydrogen bonds within the binding pocket (Fig. 3C). While docking predicted the carboxylic acid of compound ZINC263392672 to insert into the phosphate-binding tunnel, forming a hydrogen bond to Val^49^, the crystal structure instead revealed hydrogen bonds to the backbone amines of Phe^156^ and Asp^157^, which we defined as the “oxyanion” subsite within the adenosine site. Interactions with this backbone-defined oxyanion subsite were also observed for many other hits from both the docking and the crystallographically screened libraries (e.g., Fig. 3F and fig. S7E).

For a set of smaller, mainly adenine-like docking hits, modeled to only occupy the adenine subsite of the targeted adenosine-binding site (Fig. 3, D and E, and fig. S7, C and D), the comparison between docked and experimental poses revealed deviations between 1.3 and 4 Å. Making these somewhat larger deviations harder to interpret was that, for several fragments, the crystallographically observed pose, e.g., ZINC331945 (RMSD, 3.97 Å; Fig. 3E) and ZINC763250 (RMSD, 3.78 Å; fig. S7D), is partially stabilized by interactions with the symmetry mate (see below).

Another group of docking hits was selected for their close mimicry of the adenosine scaffold (Fig. 3, F and G, and fig. S7, I to L). For these, the ultrahigh resolution of the crystal structures was crucial, revealing that for four of these, the wrong purine isomer had been inadvertently synthesized, with alkyl derivatives from the N3 rather than the intended N9 nitrogen corresponding to the alkylation of adenine in ADPr (fig. S7, I to L). Characterization of the original compound samples by high-performance liquid chromatography–mass spectrometry (HPLC-MS) and nuclear magnetic resonance confirmed that the delivered compounds were >95% pure, misassigned positional isomers. For ZINC901381520 (Fig. 3F), both N3 (PDB 5RSK) and N9 (PDB 5S6W) isomers were synthesized in different batches and confirmed to bind to the targeted adenosine-binding site forming equal hydrogen bond interactions with the protein (fig. S7I). ZINC3888754 (PDB 5RSC) (Fig. 3G) contains an adenine-like heterocycle extended by methyl groups at the C7 and C8 positions, revealing opportunities for expanding purine scaffolds beyond the adenine subsite to achieve Mac1 selectivity over other adenine-binding proteins.

In addition to hydrogen bonding with residues involved in the adenine recognition of ADPr, several docking hits hydrogen bond to the backbone carbonyl group of Ala^154^ (Fig. 3, D and I, and fig. S7G), revealing an intriguing accessory polar contact within this subsite. While most residues surrounding the adenosine-binding site adopted similar conformations in the fragment-bound crystal structures, as in the ADPr-bound structure used for docking (PDB 6W02) (*34*), Asp^22^ and Phe^156^ adopted multiple, alternative conformations. In most fragment-bound crystal structures, Phe^156^ rotated by approximately 90°, enabling improved face-to-face π-π stacking against the aromatic moieties in the bound fragments (Fig. 3, C to G). However, the docking template orientation of Phe^156^ was retained for other pyrimidine-containing fragment-bound crystal structures (Fig. 3, B and H).

Overall, two characteristics stand out from the docking screen: First, despite some important differences, there was high fidelity between the docking-predicted poses and those observed by crystallography. The docking hits explored the adenine subsite to which they were targeted. Second, these hits did so with relatively dense variations around several chemotypes, something afforded by the granularity of a >20 million–fragment library. This density can be explored further, for example, 9170 fragments (888 unique BM scaffolds) in the ZINC15 fragment library contained 7*H*-pyrrolo(2,3-*d*)pyrimidines; the functional group repeatedly observed in crystallographically confirmed docking hits (Fig. 3, A to C).

### Analysis of key interactions between Mac1 and fragments from the crystallographic screens

#### Fragments binding to the adenine subsite

**Fig. 4:**
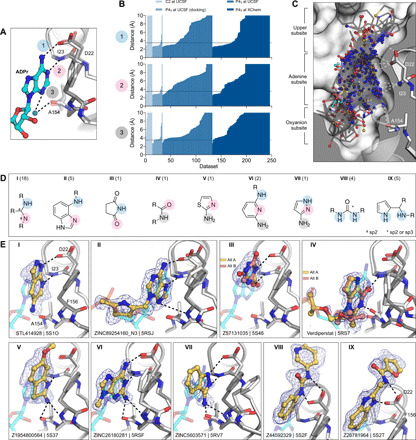
Fragments binding to the adenine subsite. (**A**) Stick representation showing the interaction of the adenosine moiety of ADPr with Mac1. The key hydrogen bonds are shown as dashed lines. (**B**) Plot of the distances shown in (A) for all fragment hits. The distances, truncated to 10 Å, are for the closest noncarbon fragment atom. (**C**) Stick representation showing all fragments interacting with Asp^22^-N, Ile^23^-N, or Ala^154^-O. The surface is “sliced” down a plane passing through Asp^22^. (**D**) Structures of the nine unique motifs that make at least two hydrogen bonds to the adenine subsite. Colored circles match the interactions listed in (A) and (B). The number of fragments identified for each motif are listed in parentheses. (**E**) Examples of the nine structural motifs. The fragment is shown with yellow sticks and the PanDDA event map is shown as a blue mesh. ADPr is shown as cyan transparent sticks. The apo structure is shown with dark gray transparent sticks.

While docking was successful in targeting the adenine-binding subsite, crystallographic fragment screening has the advantages of being binding site agnostic and has the potential to identify novel chemotypes at multiple sites. In total, crystallographic screening identified 99 fragments that form subsets of the three hydrogen bonds found between Mac1 and ADPr within the adenine subsite (Fig. 4, A to C). Fragments that formed at least two hydrogen bonds to the adenine subsite were separated into nine classes based on the number, nature, and connectivity of atoms involved in this hydrogen bonding (Fig. 4D). The most common class consisted of a 1,3-hydrogen bond donor/acceptor motif (Fig. 4, D, E, and I). This resembles the kinase hinge–binding motif, with the difference being the engagement of a side-chain oxygen rather than a backbone carbonyl oxygen (fig. S8, A and B) (*43*). While 7 of 18 fragments in this class were 4-amino-pyrimidine derivatives, other moieties were also found, including two 2-amino-thiazole–based fragments and several purine derivatives (data file S1). We also observed an unusual adenine-binding mode with a hydrogen bond formed between Ile^23^ and N7 instead of N1 (Fig. 4, D and E, II). The alternative binding mode can be explained by the N3 substitution of adenine on this fragment, which prevents formation of the canonical N1-Ile^23^ hydrogen bond. This pattern of hydrogen bonds to the protein has not been previously observed in adenines linked through N9 (*44*).

We also observed diverse fragments without adenine-like motifs binding at this site, including succinimides, amides, thiazoles, diaminopyridines, pyrazoles, pyrroles, and ureas (Fig. 4, D and E, III to VIII). These exploited, separately and together, Asp^22^, Ile^23^, Ala^154^, and, occasionally, all three adenine-defining hydrogen-bonding residues. Several fragments π-π stacked with Phe^156^, while those bearing a urea hydrogen bonded with the carboxylate of Asp^22^ (Fig. 4, D and E, VIII). These interactions were reproduced by a series of benzimidazole-based fragments (Fig. 4, D and E, IX). These classes occupied what might be classified as an “upper” subsite, above that defined by the adenine-ribose axis (Fig. 2G), and may provide an opportunity to grow fragments away from the canonical site.

#### Fragments binding to the oxyanion subsite

**Fig. 5:**
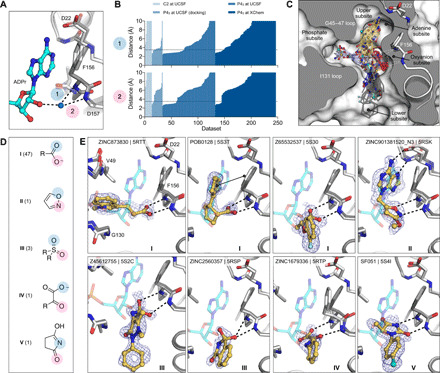
Fragments binding to the oxyanion subsite. (**A**) Stick representation showing the interaction of ADPr with the oxyanion subsite of Mac1. The water molecule bridging the ribose moiety and the oxyanion subsite is shown as a blue sphere. (**B**) Plot of the distances highlighted in (A) for all fragment hits. Distances were calculated as described for Fig. 4B. (**C**) Stick representation showing all fragments interacting with Phe^156^-N and Asp^157^-N. Fragments are colored by secondary binding site with blue as phosphate, black as lower, and yellow as adenine. The surface is sliced across a plane passing through Phe^156^ (white surface and gray interior). (**D**) Structures of the five structural motifs that bind the oxyanion site. (**E**) Examples of the five motifs. Three examples of motif I are shown, where the fragment also interacts with the phosphate, adenine, or lower subsite. The fragment is shown with yellow sticks, and the PanDDA event map is shown for reference as a blue mesh. ADPr is shown with transparent cyan sticks. The apo structure is shown with transparent gray sticks.

In total, we identified 54 fragments that formed interactions with an unexpected oxyanion subsite, defined by the backbone nitrogens of Phe^156^ and Asp^157^ adjacent to the adenine subsite (Figs. 2G and 5A). As suggested by its name, most of these fragments (48 of 54) were anionic (data file S1). The defining backbone nitrogens adopted a similar orientation to those defining the classic oxyanion hole of serine hydrolases such as acetylcholinesterase (fig. S8, D to F). In the Mac1-ADPr structure, the C2 hydroxyl (2′OH) of the proximal ribose interacts with the oxyanion subsite via a bridging water (Fig. 5A). In total, 54 fragments formed at least one hydrogen bond to the oxyanion subsite (Fig. 5B). Here too, the fragments were both geometrically (Fig. 5C) and chemically diverse (Fig. 5D): orienting groups either toward the phosphate tunnel, the lower site, or wrapped around toward the upper adenine subsite, providing multiple opportunities for further elaboration. Chemically, they interacted with the site using not only a carboxylate but also sulfones, as well as isoxazole, α-keto acid, and a succinimide (Fig. 5E). We suspect that the presence of the oxyanion subsite explains the higher hit rate for the Enamine Essential library versus the other crystallographic fragment libraries screened (27% versus 6%), as the former had a greater proportion of acids than the others (41% versus 4%) (fig. S5).

#### Fragments binding to the catalytic and other potential allosteric sites

**Fig. 6:**
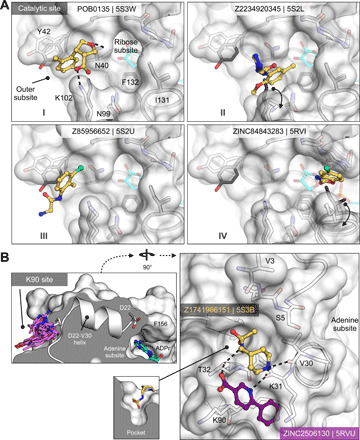
Fragments targeting the catalytic and potential allosteric sites are sparsely populated compared to the adenosine site. (**A**) Surface representation showing fragments that bind near the catalytic site. The fragment POB0135 (PDB 5S3W) bridges the gap between Asn^40^ and Lys^102^ via a hydrogen bond and a salt bridge, respectively. Although eight fragments bind in the outer subsite, the fragment POB0135 makes the highest-quality interactions. No fragments bind in the ribose subsite. The fragment ZINC331715 (PDB 5RVI) inserts into the phosphate subsite between Ile^131^ and Gly^47^. (**B**) Left: The K90 site is connected to the adenosine site by the Asp^22^-Val^30^ α helix. Right: Surface representation showing two fragments that bind to the K90 site. Hydrogen bonds are shown as dashed black lines. The fragment Z1741966151 (PDB 5S3B) is partially inserted in a nearby pocket (inset).

There were substantially fewer hits against the catalytic site (Fig. 2G) versus the adenosine site (8 versus >100), although both appear to be accessible (fig. S3B). The catalytic site consists of three subsites: the phosphate tunnel, which is occupied by the diphosphate of ADPr; the ribose subsite, which is occupied by the terminal ribose of the molecule; and the outer subsite, which sits between Asn^40^ and Asn^99^ (Figs. 2G and 6A). Of the eight fragments binding in the catalytic site, seven bound in the outer subsite, and one bound in the phosphate tunnel. Binding to the outer site was often defined by hydrophobic packing between the Tyr^42^ and Lys^102^ side chains, although POB0135 (PDB 5S3W) and POB0128 (PDB 5S3T) formed a salt bridge to Lys^102^ (e.g., Fig. 6A, I). The latter fragment was also found to bind in the adenosine site. Other molecules, including Z2234920345 (PDB 5S2L) and Z955123498 (PDB 5S4A) stabilize an alternative conformation of Lys^102^ (Fig. 6A, II). Three of the fragments, including Z85956652 (PDB 5S2U), positioned a halogen atom in the outer subsite (e.g., Fig. 6A, III). The only fragment identified in the phosphate subsite was ZINC84843283 (PDB 5RVI). This fragment was wedged between the Gly^47^/Ile^131^ loops and increased the gap between the two loops by 1.6 Å (Fig. 6A, IV). The absence of fragments binding to the ribose subsite, as well as the sparsity of fragments in the phosphate tunnel, means that designing a Mac1 inhibitor to occupy the catalytic site will rely more heavily on fragment growing than on fragment merging.

Both crystallographic screens also identified fragments binding to the K90 site, which is formed by a cleft between Lys^31^, Thr^32^, and Lys^90^ (Fig. 6B). We identified seven fragments from the C2 crystal form and six from the P4_3_ crystal form; none of the C2-derived fragments were found again when the UCSF libraries were rescreened under the P4_3_ crystal condition. Although the K90 site is 15 Å from the adenosine site, it is connected to that subsite via a single α helix (Fig. 6B). Although there is no biochemical evidence for allosteric communication between these sites, the fragments provide starting points for designing chemical probes to test this possibility.

### Fragment binding exploits protein conformational flexibility

**Fig. 7:**
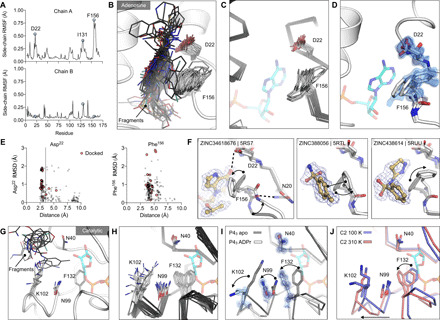
Experimentally observed conformational heterogeneity is sampled by various fragments. (**A**) Plots of side-chain RMSF for the 117 fragment structures from the UCSF screen using P4_3_ crystals. (**B**) Stick representation showing all fragments (black sticks) within 3.5 Å of the Asp^22^ carboxylate and 4 Å of the Phe^156^ ring (white sticks). (**C**) Structural heterogeneity in the previously reported Mac1 structures. (**D**) The Phe^156^ side chain is captured in three conformations in the C2 apo structure. Electron density maps (2mF_O_-DF_C_) are contoured at 0.5 σ (blue surface) and 1 σ (blue mesh). For reference, ADPr is shown with blue sticks. (**E**) Plots of side-chain RMSD for Asp^22^ and Phe^156^ from the Mac1 apo structure as a function of ligand-protein distance. Structures were aligned by their Cα atoms, before RMSDs were calculated for the Asp^22^ carboxylate and the Phe^156^ aromatic carbons. (**F**) Fragment binding exploits preexisting conformational heterogeneity in the Phe^156^ side chain. The apo structure is shown with dark transparent gray sticks in each panel, and the conformational changes are annotated with arrows. (**G**) Stick representation showing all fragments (black sticks) in the outer subsite of the catalytic site. (**H**) Conformational heterogeneity of residues in the catalytic site of the previously reported Mac1 crystal structures. (**I**) ADPr binding induces a coupled conformational change in the Phe^132^, Asn^99^, and Lys^102^ side chains, as well as a 2-Å shift in the Phe^132^ loop. Electron density maps (2mF_O_-DF_C_) are contoured at 1.5 σ (blue surface) and 4 σ (blue mesh). (**J**) Mac1 structures determined at 100 and 310 K using C2 crystals.

To identify Mac1 flexibility associated with molecular recognition, we calculated the root mean square fluctuation (RMSF) of side-chain atoms across the P4_3_ fragment-bound structures. Residues lining the adenosine site, especially Asp^22^ and Phe^156^, are the most flexible (Fig. 7, A and B). The flexibility of both residues is paralleled in previously reported crystal structures (Fig. 7C) and also in the 0.77-Å apo (apoenzyme) structure, where multiple alternative conformations are clearly defined in electron density maps (Fig. 7D and fig. S1, A to C). In the ultrahigh-resolution structure, residues 155 to 159 are modeled as a combination of two distinct backbone conformations that diverge substantially at Phe^156^, which requires three distinct conformations of this residue to satisfy the observed density (Fig. 7D and fig. S1C). Despite this flexibility, hydrogen bonds to Asp^22^ are present in many fragments, including docking compounds that were chosen on the basis of interactions with a static receptor (Fig. 7E). Similarly, the flexibility of the aromatic side chain of Phe^156^ enables adaptable stacking interactions with fragments (Fig. 7, E and F), with 46 fragments binding within 4 Å of Phe^156^. As with Asp^22^, the nature and geometry of these interactions are maintained for many soaked and docked fragments even as the residue moves relative to the rest of the protein.

In contrast to the adenosine site, little conformational heterogeneity is observed at the catalytic site, with only minimal changes in Lys^102^ and Tyr^42^ conformations (Fig. 7G). Still, even in this site, there is more conformational heterogeneity observed in previously published structures (Fig. 7H). In particular, a network of flexible side chains encompassing Phe^132^, Asn^99^, and Lys^102^ is stabilized in a distinct conformation upon ADPr binding (Fig. 7I). To further probe the flexibility of the Phe^132^-Asn^99^-Lys^102^ network, we determined structures of Mac1 using the C2 crystal at human physiological temperature (37°C, 310 K) to 1.5-Å resolution (Fig. 7J and data file S1). As observed in other systems (*45*, *46*), we noticed that the cryogenic structure appeared more compact than the structure at higher temperatures. Specifically, we observed substantial loop displacements near the ribose-binding pocket of the active site, which are coupled to a global hinge-bending motion involving correlated motion of helices about the central β sheet (fig. S4, F and G). The structure at physiological temperature more closely resembles the structure with ADPr bound, with the backbone adopting a more open conformation (Fig. 7J). However, the side-chain rotamers of Asn^99^ and Lys^102^ do not undergo the larger rearrangements. This temperature-dependent change in the width of the active site cleft can provide alternative, potentially more relevant, conformations for future ligand discovery efforts targeting the catalytic site around the distal ribose.

### Changes in water networks upon fragment binding

**Fig. 8:**
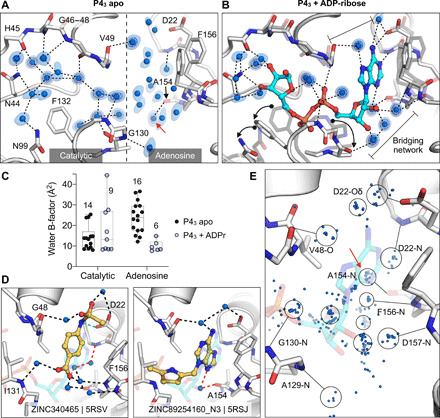
Water networks in the active site are displaced and used by fragments for bridging interactions. (**A**) Water networks in the apo enzyme (P4_3_ crystal form). Waters are shown as blue spheres, with electron density contoured at 5.0 σ (blue mesh) and 1.5 σ (blue surface). Hydrogen bonds are shown as dashed lines (distances are 2.6 to 3 Å). (**B**) Water networks in the Mac1-ADPr complex. ADPr is shown as cyan sticks. Conformational changes upon ADPr binding are highlighted with black arrows. (**C**) Comparison of crystallographic B-factors of water molecules in the catalytic site and adenosine site. The range and 95% confidence interval are shown. (**D**) Examples of the role of water networks in fragment binding. Left: ZINC340465 (PDB 5RSV) forms a single hydrogen bond to the protein (green dashed line) but forms five hydrogen bonds via water molecules. Right: Although few fragments of hydrogen bond directly to the backbone oxygen of Ala^154^, several fragments interact with this residue via bridging water molecules (red dashed line) including ZINC89254160_N3 (PDB 5RSJ). (**E**) Plot showing all water molecules that lie within 3.5 Å of a noncarbon fragment atom. Water molecules are shown as blue spheres, with the major clusters circled. The cluster highlighted with a red arrow bridges fragments and the Ala^154^ backbone oxygen.

To assess the role of water networks in fragment binding, we first examined changes in water networks upon ADPr binding. In the 0.85-Å P4_3_ apo structure, the catalytic site contains 14 water molecules arranged in an ordered network that connects the Gly^47^ loop and the Ile^131^ loop, with an arc formed around the Phe^132^ side chain (Fig. 8A). In contrast, waters were more disordered in the adenosine site, with more diffuse electron density and higher B-factors (Fig. 8, A and C). Upon ADPr binding, five waters were displaced from the catalytic site, and the water network was disrupted (Fig. 8B). This disruption is partly caused by altered conformation of the Phe^132^ and Asn^99^ side chains, which break the network between residues Asn^40^ and Asn^99^. Conversely, the network in the adenosine site was stabilized in the Mac1-ADPr complex (Fig. 8B). The average B-factor decreased from 24 to 10 A^2^, and two networks connect the phosphate tunnel with the adenine/oxyanion subsites (Fig. 8C). Although the adenine moiety only forms two direct hydrogen bonds to protein, it has four additional contacts via bridging water molecules (Fig. 8B). Similar bridging waters were observed for fragments binding in the adenosine site including ZINC340465 (PDB 5RSV), which forms only one direct hydrogen bond to the protein but has an extensive hydrogen bond network via water molecules (Fig. 8D). Visualizing all water molecules within 3.5 Å of fragment atoms shows clusters near protein hydrogen bond acceptors and donors (Fig. 8E). Of particular interest is the cluster near the backbone carbonyl of Ala^154^. This site is occupied by a water molecule in the Mac1-ADPr structure and is bridged by adenine derivatives such as ZINC340465 (PDB 5RSJ) (Fig. 8D). In addition, five fragments occupy this site directly (Fig. 4, A and D), including the C2-amino–substituted adenine present in ZINC89254160_N3 (PDB 5RSJ; Fig. 3D). Extending fragments to displace the water molecules at other frequently populated sites could help to quantify the contribution of water networks to Mac1 binding and to provide a test set for computational methods that seek to exploit solvent dynamics for ligand optimization (*47*, *48*).

### Solution binding of fragment hits

**Fig. 9:**
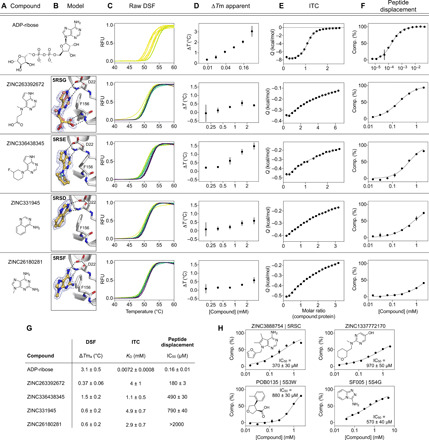
Biophysical corroboration of solution binding of crystallographic fragment hits by DSF, ITC, and ADPr-peptide displacement assay. Top: (**A** to **F**) Performance of the most potent fragment hits in DSF, ITC, and ADPr-peptide displacement assay compared to ADPr. (C) Normalized raw DSF relative fluorescence unit (RFU) data demonstrate canonical unfolding curves and minimal compound-associated curve shape aberrations. Gradient color scales, 0 mM (yellow) and 3 mM (purple). (D) *T*m_a_ elevation reveals Mac1 stabilization through fragment binding. Data points represent the means ± SD for triplicate measurements at each compound concentration. (E) Integrated heat peaks measured by ITC as a function of compound:protein molar ratio. The black line represents a nonlinear fit using a single-site binding model. (F) Peptide displacement assay measures ADPr-peptide displacement (i.e., % competition) from Mac1 by ligand. Data points represent the means ± SD for duplicate measurements at each compound concentration, and the black line represents a nonlinear fit using a sigmoidal dose-response equation constrained to 0 and 100% competition. (**G**) Summary of solution binding data for fragments from top panels. Δ*T*m_a_ values are given for the highest compound concentration in this assay (means ± SD). For the ITC and peptide displacement experiments, parameters obtained by nonlinear regression are given (±estimated SE). (**H**) Additional fragment hits showing Mac1 peptide competition.

To buttress the crystallographic studies, we biophysically screened selected compounds using DSF, ITC, and an HTRF ADPr-peptide displacement assay (Fig. 9 and data files S1 and S2). Because of their ready availability in useful amounts, most of these experiments focused on the docking hits. For DSF, in agreement with previous reports for this enzyme (*18*), we observed substantial elevation of the apparent melting temperature (*T*m_a_) upon addition of ADPr (Fig. 9, C, D, and G). When tested in concentration response from 0.188 to 3 mM, 10 of 54 docked fragments also induced small but statistically significant and dose-responsive *T*m_a_ elevation (Fig. 9, C, D, and G, and data file S1). All 10 of these were also observed to bind in the crystallographic studies, providing relatively good agreement between these assays. However, the correlation was incomplete, as the remaining fragments observed by crystallography either decreased the *T*m_a_ or had no significant effect (data file S1).

To identify fragments with the most promising binding affinity for optimization, we tested the 19 crystallographically observed docking hits using ITC (data files S1 and S2). Because of their small size, most of these fragments have low binding affinity and release little heat upon binding versus ADPr. Thus, we only observed reliable thermodynamic measurements for 4 of the 19 fragments. These could be fit to a single-site binding model with affinities in the low millimolar range (Fig. 9, E and G), consistent with the DSF results. Furthermore, the compounds measured by ITC that released the greatest amount of heat also induced significant *T*m_a_ shifts in DSF (data file S1).

Last, we tested 57 docking-derived fragments and 18 crystallographic hits from the XChem library in an HTRF-based peptide displacement assay, which monitors displacement of a fluorescently labeled ADPr-conjugated peptide from the active site of Mac1 (Fig. 9, F and G, and data files S1 and S2). Eight of 57 docking hits (14%) and 3 of 18 crystallographic hits (17%) inhibited the enzyme with median inhibitory concentration (IC_50_) values between 180 μM and 1 mM, with the most potent fragment being the docking-derived ZINC263392672 (PDB 5RSG) with an IC_50_ of 183 μM in this assay. Only 5 [ZINC3888754 (PDB 5RSC), ZINC331945 (PDB 5RSD), ZINC263392672 (PDB 5RSG), ZINC336438345 (PDB 5RSE), and ZINC6180281 (PDB 5RSF); Fig. 3] of the 10 docking hits that stabilized Mac1 as measured by DSF were inhibitory in the ADPr-peptide displacement assay. Two docking hits that were not identified as binders by DSF or crystallography, ZINC1337772170 (IC_50_ = 971 μM) and pterin (IC_50_ = 784 μM), were found to be inhibitors in the peptide displacement assay (Fig. 9H). This result might be explained by the use of a detergent in the peptide displacement assay that could increase compound solubility. With its ability to detect inhibition of Mac1, the ADPr-peptide displacement assay proved to be a sensitive and complementary strategy for further characterization of the fragment hits obtained from the docking and crystallographic screens. Assuming that the HTRF-based peptide displacement assay produced the most reliable inhibition data, we estimated ligand efficiencies from IC_50_ values for hits for which we obtained reasonable dose-response curves. ADPr, with an IC_50_ of 161 nM and 36 heavy atoms, has an LE of 0.26 kcal/mol per nonhydrogen atom. The docking hits ZINC3888754 (PDB 5RSC, LE = 0.26), ZINC336438345 (PDB 5RSE, LE = 0.28), ZINC263392672 (PDB 5RSG, LE = 0.32), and ZINC331945 (PDB 5RSD, LE = 0.38) reveal similar or slightly improved ligand efficiencies, while the highest LE was calculated for the XChem library hit SF005 (PDB 5S4G; Fig. 9H), with 0.44 kcal/mol per heavy atom.

In summary, all crystallographically confirmed docking hits were tested using three complementary in-solution binding techniques, DSF, ITC, and an HTRF-based peptide displacement assay (fig. S9 and data file S1). ZINC336438345 (PDB 5RSE), ZINC331945 (PDB 5RSD), ZINC263392672 (PDB 5RSG), and ZINC26180281 (PDB 5RSF) were the only four fragment hits for which binding data could be obtained by all three techniques (Fig. 9). All of these fragments have key hydrogen bonds in the adenine subsite and π-π stack with Phe^156^. Furthermore, ZINC263392672 (PDB 5RSG) interacts via its carboxyl group with the oxyanion subsite of Mac1. Last, we note that crystallography, DSF, and ITC all monitor binding but do not measure function. The peptide displacement assay is thus of particular value for fragment characterization because it measures specific displacement of an analog of the natural Mac1 substrate.

### Opportunities for fragment linking and merging to optimize Mac1 inhibitors

**Fig. 10:**
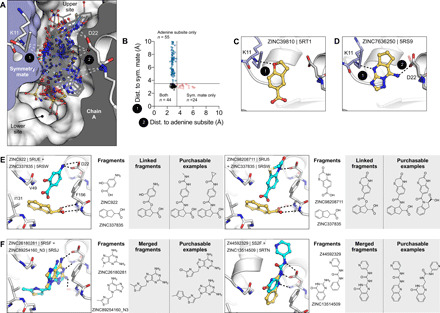
Fragments bridging multiple adenosine sites provide direct merging opportunities. (**A**) Sliced view of the adenosine site (white surface and gray interior) and a symmetry mate (blue surface and interior) showing the deep pocket created by crystal packing in the P4_3_ crystals. The 66 fragments that hydrogen bond with the Lys^11^ backbone nitrogen are shown as sticks. (**B**) Plot showing distances between the symmetry mate (Lys^11^-N) and the adenine subsite (Asp^22^-Oδ, Ile^23^-N, and Ala^154^-O) for all fragments identified in the adenosine site. Dotted lines show the 3.5-Å cutoff used to classify hydrogen bonds. (**C**) An example showing 1 of the 24 fragments that bound in the adenosine site, yet only formed a hydrogen bond with the symmetry mate. (**D**) An example of one of the fragments that bridged the 9- to 11-Å gap between the adenine subsite and the symmetry mate. (**E** and **F**) Opportunities for fragment linking and merging. Adjacent or overlapping fragments were initially merged into a single new compound. Examples of readily available make-on-demand compounds are shown.

Typically, one might be reluctant to speculate on optimization from fragment structures alone, but the unusually large number of structures perhaps supports some cautious inference here. Before modifying, linking, or merging fragments, it is important to consider the crystalline environment. In the P4_3_ crystal form, the active site forms a bipartite-enclosed pocket with a symmetry mate (Fig. 10, A and B). In particular, 24 fragments only hydrogen bond to Lys^11^ of the symmetry mate and not with any residues in the adenosine site, indicating that these molecules should not be considered for fragment elaboration (Fig. 10, C and D). On the basis of the binding poses of remaining compounds, fragment pairs were linked into hypothetical scaffolds. These were used as templates to search the make-on-demand chemical space of the Enamine REAL database using the SmallWorld similarity (http://sw.docking.org) and Arthor substructure (http://arthor.docking.org) search engines (Fig. 10, E and F) (*49*). In a second approach, fragments with overlapping binding poses were merged into larger scaffolds, e.g., the purine of ZINC89254160_N3 (PDB 5RSJ) interacting in the adenine-binding subsite was replaced by ZINC26180281 (PDB 5RSF), adding an additional hydrogen bond to Ala^154^ (Fig. 10F). Whereas it remains speculative whether the suggested linked or merged molecules are indeed active against Mac1, the scaffolds observed here, as well as the key interactions they make with the enzyme, indicate a fruitful chemical space to further explore. Naturally, many of the fragments described here also merit investigation by alternative fragment growing or analoging strategies.

## DISCUSSION

Three key observations emerge from this study. Most noteworthy is the sheer number and the unusually high resolution of the 234 fragment-bound Mac1 structures, including 192 fragments identified in the active site. The fragments cover both stereotypical interactions (such as adenine-like hydrogen bonding to the Asp^22^ side chain/Ile^23^ backbone and stacking interaction with Phe^156^) as well as diverse and unusual chemotypes that exploit active site flexibility (for instance, by targeting the oxyanion subsite). This abundance and diversity afford multiple starting points for future elaboration into lead-like molecules. Second, the high fidelity of docked poses to the subsequent crystallographic results supports the use of docking to explore the adenine recognition site and demonstrates an ability of docking to prioritize fragments, at least for this target, something still debated in the field. Last, with 234 diverse fragment structures determined, it should be possible to exploit the fortuitous juxtaposition of fragment pairs to design joined ligands that combine the affinities of both, leading to inhibitors with the low micromolar affinity needed for hit-to-lead optimization. One clear strategy involves extending molecules bound to the adenine subsite and with biophysically measurable binding affinities into the phosphate and distal ribose recognition regions.

In contrast to the large number of chemically diverse hits binding to the adenine subsite, the lack of fragments bound to the catalytic site is notable and may inform models of how ADP-ribosylated peptides bind to Mac1. The paucity of fragments is especially unusual given that three crystal environments (the A and B chains in the P4_3_ crystal and the C2 crystal) were screened and that the site appears accessible in all lattices (fig. S2B). The two major models for peptide-macrodomain interactions are either that the peptide binds along the widened cleft defined by Tyr^42^ and Lys^102^ or that it extends into solution through the flexible Gly^46–48^ loop (*50*). We observe fragments that bind in both locations (Fig. 6A). Regardless of the binding mode, which could be distinct depending on the identity of the modified residue and target substrate, the lack of binding at this site suggests that the binding energy comes mostly from the ADPr and not from the amino acids on the ADPr-conjugated protein. This hypothesis is also supported by the fact that Mac1 can hydrolyse a wide range of ADP-ribosylated substrates (*2*, *51*). Docking of larger “lead-like” molecules, perhaps enabled by the expanded catalytic site revealed by the physiological temperature structure and detailed description of solvent, may help to identify molecules exploiting this site.

The success of the fragment docking campaign contrasts, perhaps, with expectations of the field that fragments have too few functional group handles to accurately dock or prioritize (*52*). Not only were hit rates high (33%) but also was the fidelity of most docking poses to the crystallographic results. Even judged by potency, the most active fragment to emerge from this study, the 183 μM inhibitor ZINC263392672 (PDB 5RSG) (Figs. 3 and 9), was a docking hit. In addition, it was the docking hits that were most readily available for this functional testing, as they were sourced in 10 mg amounts, while the crystallographic screening compounds were often in short supply. This is a purely mechanical advantage of docking, and it is counterbalanced by the small numbers tested versus the crystallographic screens; still, having substantial material to work with is a pragmatic advantage. Admittedly, weaknesses also emerged from the docking. The oxyanion site that featured so prominently among the crystallographic screening hits was not to be found among the docking predictions. This gap reflects both a failure of the docking scoring function to prioritize anions binding to this site (as they were at least sampled) and, to some extent, a failure of the docking group to pick the few molecules that did dock well to this site as likely candidates. More broadly, as we docked against a single rigid structure of the protein, the subsequent conformational changes that the protein underwent, as well as the changes in the water network, were not captured in the docking predictions, and this was sometimes reflected in the larger RMSD differences between predicted and observed fragment poses (Fig. 3). These caveats, important as they are, should not obscure a central observation from this study: The docking hit rate was not only high, but the hits were also typically right for the right reasons; this may be something to build on for the field.

From the docked compounds, the most promising hits identified by in-solution binding experiments were also crystallographically confirmed. However, as expected, most hits from crystallography did not show appreciable activity in the orthogonal biophysical assays within the tested concentration range (up to 10 mM in ITC; data file S1). The macrodomain ADPr-peptide displacement assay also identified two docking hits not previously observed in soaking (ZINC1337772170 and pterin), which suggests that the crystal environment limited the ability of some fragments to bind. However, between solution experiments, good consensus was observed for ZINC263392672 (PDB 5RSG), ZINC336438345 (PDB 5RSE), and ZINC331945 (PDB 5RSD). While we are aware that obtaining high-quality binding data remains particularly challenging for weak binders such as fragments, the dose-response results obtained in the complementary assays for many of the identified hits provided convincing evidence for their true binding to Mac1. The inconsistency of fragment binding to different crystal systems of the same protein is apparent when comparing fragments that resulted in high-quality datasets in both the P4_3_ and C2 crystal systems. Unexpectedly, only 5 of 59 possible fragments were observed in both systems, with 3 fragments binding with equivalent poses in the adenine subsite. This observation points to the value of having multiple measurements, and even multiple crystal systems when they are available, in fragment-based drug discovery approaches.

Overall, this study has three main implications for the discovery of SARS-CoV-2 Nsp3 Mac1 inhibitors and for antiviral efforts targeting macrodomains more broadly. First, we not only describe new chemical matter for this target but also map its hotspots at high resolution. This provides a template for future inhibitor discovery and development against this enzyme. These efforts will need to navigate selectivity over human macrodomains and other adenosine triphosphate–binding proteins including kinases (fig. S8, A to C) and consider breadth across other viral macrodomains (fig. S4, I and J) (*12*). Second, the specific fragments that we describe may lend themselves directly to optimization: Several examples are discussed explicitly, amenable to make-on-demand chemistry (Fig. 10, E and F), and the 234 structures should provide inspiration for countless other molecules. Last, important technical advances emerged from this study: a crystal form that lends itself to ready structure determination, the creation of a reliable peptide displacement assay for Mac1, and evidence supporting the ability of structure-based screening, such as molecular docking, to predict effective fragments. The ultrahigh-resolution x-ray diffraction data, which allowed hydrogen atoms to be refined explicitly, as well as electron density to be resolved on a subatomic scale, make Mac1 an attractive candidate for in-depth computational dissection of its catalytic mechanism using approaches that integrate both classical and quantum calculations. Together, these advances will speed progress throughout the community to help validate this target and create effective antivirals.

## MATERIALS AND METHODS

### Fragment libraries

We screened 2122 molecules from the XChem facility at Diamond Light Source against the Mac1 P4_3_ crystal form and 411 molecules from UCSF against the C2 and P4_3_ crystal forms (data file S1). The fragment library at XChem combined molecules from multiple fragment libraries: the Diamond Light Source, SGC, and iNEXT (DSI)–poised library [687 molecules (*36*)], the Edelris fragment collection (132 molecules), the MiniFrags Probing Library [80 molecules (*53*)], the FragLites collection [31 compounds (*54*)], the PepLite library [22 molecules (*26*)], the SpotFinder library (96 compounds), the York3D library [106 molecules (*55*)], and the EU-OPENSCREEN (968 molecules). The UCSF fragment library was composed of Enamine’s Essential Fragment library (320 compounds) and 91 additional compounds from an in-house library (UCSF_91). To assemble the UCSF_91 library, we selected topologically diverse molecules having more than 10,000 commercially available analogs in at least three points of substitution, allowing for rapid and extensive analog by catalog without having to resort to flask synthesis. We picked molecules that were also BM scaffolds (*38*), stripped of acyclic terminal substituents. We thought simple, unsubstituted frameworks would be easier to optimize by adding chemical matter during analoging. From among these, we prioritized by eye scaffolds with various ring sizes and combinations including fused rings, spiro systems, with linkers of varying lengths between rings, in an attempt to sample a diverse range of compact shapes and properties. We added anions where the anionic moiety was a small acyclic substituent on the scaffold, again picking by eye for shape diversity. We chose molecules with 11 to 21 heavy atoms, with molecular weights between 200 and 300 amu and with a log*P* < 2.5 for solubility. Physical properties of all screened libraries are shown in fig. S5.

Analyses of scaffolds and specific chemotypes in the used chemical libraries are shown in fig. S5E. BM scaffold analysis was performed with the Molinspiration mib engine (www.molinspiration.com). Pyrimidines were identified using RDKit (www.rdkit.org), and molecular charges at pH 7.4 were approximated using ChemAxon JChem version 2019.15 (www.chemaxon.com) to identify anionic fragments.

### C2 crystals at UCSF

#### Protein expression and purification

SARS-CoV-2 Nsp3 Mac1 (residues 2 to 170) was cloned into a pET22b(+) expression vector with an N-terminal His_6_ tag and a tobacco etch virus (TEV) protease recognition site for removal of the tag (GenScript). In addition, a short linker (Asn-Ala-Gly) was included between the TEV recognition site and the Mac1 gene (data file S1). To express Mac1, plasmid DNA was transformed into BL21(DE3) *Escherichia coli*. After overnight growth on lysogeny broth (LB) agar supplemented with carbenicillin (100 μg/ml), starter cultures (10 ml of LB) were grown at 37°C for 8 hours. Large-scale cultures [1 liter of terrific broth] were grown at 37°C until an optical density of 0.8. Cultures were cooled at 4°C for 15 min, before protein expression was induced with 1 mM isopropyl β-d-1-thiogalactopyranoside (IPTG), and the cultures were shaken at 20°C for 12 hours. Cells were collected by centrifugation and frozen at −80°C.

All purification steps were performed at 4°C using an AKTA fast protein liquid chromatography system (Cytiva). Cells were resuspended in Ni–nitrilotriacetic acid (NTA) binding buffer [50 mM tris-HCl (pH 8.0), 500 mM NaCl, 10 mM imidazole, 5% glycerol, and 2 mM β-mercaptoethanol (βME) supplemented with TurboNuclease (5 U/ml; Sigma-Aldrich, T4330)] and lysed by sonication. Cell debris was collected by centrifugation, and the lysate was applied to a 5-ml HisTrap HP column (Cytiva, 17524802). The column was washed with 25 ml of binding buffer followed by 25 ml of 5% Ni-NTA elution buffer [50 mM tris-HCl (pH 8.0), 500 mM NaCl, 500 mM imidazole, 5% glycerol, and 2 mM βME] and then eluted with 100% elution buffer. Eluted protein was exchanged into TEV reaction buffer [50 mM tris (pH 8.0), 100 mM NaCl, 1 mM dithiothreitol (DTT), and 1% glycerol] using a HiPrep 26/10 desalting column (Cytiva, 17508701). To cleave the His_6_ tag, Mac1 was diluted to 1.5 mg/ml using TEV reaction buffer and incubated with recombinant TEV protease (*56*) at a 1:20 ratio (Mac1:TEV) for 16 hours at 4°C. Cleaved Mac1 was separated from the uncleaved protein and TEV protease by rerunning the sample over a HisTrap HP column (pre-equilibrated with TEV reaction buffer) and collecting the flow-through. The flow-through was supplemented with 10 mM DTT and concentrated to 2.5 ml using a 10-kDa molecular weight cutoff (MWCO) centrifugal concentrator (Amicon, UFC901024). The sample was further purified by size exclusion chromatography (SEC) using a HiLoad 16/600 Superdex 75 pg column (Cytiva, 28989333) equilibrated with SEC buffer [20 mM tris (pH 8.0), 150 mM NaCl, 5% glycerol, and 2 mM DTT]. Eluted fractions were concentrated to 15 mg/ml, flash-frozen in liquid nitrogen, and stored at −80°C. Protein used for ITC was purified in the same manner, but the SEC was run with 150 mM NaCl and 20 mM tris (pH 8.0). Protein was concentrated to 10.8 mg/ml before flash-freezing in liquid nitrogen and storage at −80°C.

#### Crystallization

Crystals were grown at 19°C using sitting-drop vapor diffusion with a reservoir solution containing 100 mM tris (pH 8.5), 100 mM sodium acetate, and 28% polyethylene glycol, molecular weight 4000 (PEG-4000). Crystallization drops were set up with 200 nl of protein and 200 nl of reservoir. Initially, crystals were grown in MRC two-well plates (SWISSCI, MRC96TUVP) with a reservoir volume of 40 μl. Crystals grew to a maximum size after 1 to 2 days and were vitrified in liquid nitrogen without additional cryoprotection. For diffraction experiments at physiological temperatures, crystals were mounted using ALS-style goniometer bases (MiTeGen, GB-B3S) and sealed with plastic capillary and vacuum grease (MiTeGen, RT-T1). The capillary contained 4 μl of reservoir solution to prevent crystal dehydration.

Fragment soaking was performed using crystals grown with SWISSCI three-well plates (SWISSCI, 3W96T-UVP). Microseeding was required to achieve consistent nucleation. Several large crystals grown in 100 mM tris (pH 8.5), 100 mM sodium acetate, and 28% PEG-4000 were transferred to a drop containing 5 μl of seed storage buffer [100 mM tris (pH 8.5), 100 mM sodium acetate, 32% PEG-4000, and 2 mM DTT] on a silicon coverslip (Hampton Research, HR3-233). Crystals were crushed using a flattened glass rod and transferred to 200 μl of seed storage buffer, before being serially diluted 1:10 with seed storage buffer. Consistent nucleation was achieved with seeds at a 1:100 dilution, with crystallization drops containing 200 nl of reservoir, 100 nl of seed stock, and 300 nl of protein with 30 μl in each reservoir.

#### Crystal dehydration and fragment soaking

Fragments were added to crystallization drops using acoustic dispensing with an Echo 650 liquid handler (Labcyte) (*23*). Two libraries were soaked at UCSF: the Enamine Essential fragment library (Enamine, 320 fragments) and the UCSF_91 library (91 fragments) (data file S1). To limit DMSO-induced crystal damage, fragments were targeted to crystallization drops as far away from crystals as possible (*23*). Initial DMSO tolerance tests indicated that the C2 crystals were sensitive, rapidly disintegrating upon soaking with 10% DMSO (fig. S2B). To enhance DMSO tolerance, 300 nl of a solution containing 35% PEG-4000, 100 mM tris (pH 8.5), and 100 mM sodium acetate was added to drops containing crystals using the Echo. Plates were resealed and incubated at 19°C for 6 hours. Fragment solutions (120 nl, 10% of the drop volume) were added using the Echo, and plates were resealed and incubated at 20°C for 3 to 8 hours. Crystals were vitrified directly from crystallization drops without additional cryoprotection.

#### Lysine methylation

Lysine methylation is a routine strategy for altering the crystallization properties of a protein (*35*). All reagents were added with the protein on ice, and incubation steps were performed at 4°C with gentle shaking. First, 20 mg of Mac1 was exchanged into lysine methylation buffer [50 mM Hepes (pH 7.5), 150 mM NaCl, and 5% glycerol] using a HiPrep 26/10 desalting column. The protein was diluted to 1 mg/ml with lysine methylation buffer, and 400 μl of 1 M dimethylamine borane (DMAB; prepared in water) (Sigma-Aldrich, 180238) and 800 μl of 1 M formaldehyde (prepared in water) (Sigma-Aldrich, F8775) were added to initiate the methylation reaction. The reaction was left to proceed for 2 hours, and then 400 μl of 1 M DMAB and 800 μl of 1 M formaldehyde were added. After an additional 2 hours, 200 μl of 1 M DMAB was added, and the reaction was left for further 16 hours. To consume any remaining formaldehyde and to cleave any intermolecular disulfide bonds, 2.5 ml of 1 M glycine (prepared in water) and 2.5 ml of 50 mM DTT (prepared in water) were added, and the reaction was incubated for an additional 2 hours. Next, the sample was concentrated to 2.5 ml using a 10-kDa MWCO concentrator and purified by SEC. The methylated protein was concentrated to 15 mg/ml before flash-freezing in liquid nitrogen and storage at −80°C.

To test the extent of lysine methylation, the purified sample was analyzed by LC-MS, using a Waters Acquity LC connected to a Waters TQ detector with electrospray ionization. The sample was separated on a C4 column held at 40°C using water with 0.1% formic acid as solvent A and acetonitrile with 0.1% formic acid as solvent B. After sample injection [5 μl at 10 μM diluted in 150 mM NaCl and 20 mM tris (pH 8.0)], an isocratic elution was run with 95% solvent A and 5% solvent B for 1.5 min. Then, a linear gradient elution was run for 6.5 min to 95% solvent B. Last, an isocratic elution was run with 95% solvent B for 2 min. The flow rate was 0.2 ml/min.

#### Crystallization of methylated Mac1

Crystals grew readily under the same conditions as the nonmethylated protein [100 mM tris (pH 8.5), 100 mM sodium acetate, and 28% PEG-4000]. Consistent nucleation was achieved using microseeding with the same protocol as the nonmethylated protein. Crystallization drops were set up with 100 nl of reservoir, 100 nl of seed stocks, and 200 nl of protein using SWISSCI three-well plates. The methylated crystals displayed increased DMSO tolerance, so DMSO/fragment soaks were performed directly with 40 nl of DMSO (10% of the drop volume).

#### Ultrahigh-resolution data collection, refinement, and modeling

To measure the diffraction at such high resolution, we used a multipass, multicrystal data collection strategy. We collected ultrahigh-resolution x-ray diffraction data for Mac1 (C2 crystal form) by performing sequential high-energy (17,000 eV) and low-energy (11,111 eV) runs to accurately measure reflection intensities at high and low scattering angles, respectively. The same data collection strategy (wedge, oscillation angle, and exposure) was implemented for multiple crystals, each held in different orientations relative to the x-ray beam and phi rotation axis.

The datasets were individually indexed and integrated with XDS (*57*). During data processing, we merged the high- and low-resolution datasets from multiple crystals in different orientations to maximize our coverage of reciprocal space given a square detector surface. A low-resolution cutoff of 2.5 Å was applied to the high-resolution (high-energy) datasets because this cutoff simultaneously excludes potentially overlapping reflections at low scattering angles and allows for a large number of shared observations between high- and low-resolution datasets, which facilitates robust scaling. Scaling and merging were performed using XSCALE, and the merged intensities were converted to structure factor magnitudes using XDSCONV (*57*).

We calculated phases by the method of molecular replacement, using the program Phaser (*58*) and a previous structure of Mac1 (PDB 6WCF) as the search model. The model was manually adjusted in Coot (*59*) to fit the electron density map calculated from molecular replacement, followed by automated refinement of coordinates, atomic displacement parameters, and occupancies using phenix.refine (*60*) with optimization of restraint weights. Following two initial rounds of iterative model building and refinement using the aforementioned strategy, we began introducing additional parameters into the model, enabled by the extraordinarily high resolution of our diffraction data. First, we implemented anisotropic atomic displacement parameters for heavy atoms (C, N, O, and S), followed by refinement of explicit hydrogen atom positions. During early rounds of model building, we noticed mF_O_-DF_C_ difference density peaks appearing between heavy-atom positions, suggesting that we are able to resolve covalent bonding densities (fig. S1E). Atomic refinement that included a model for interatomic scatterers (IASs) (*61*) was able to account for these densities and reduce the free *R* value by approximately 0.0043 (0.43%). Although the refined atomic coordinates do not differ substantially based on the inclusion or exclusion of IASs, the maximum likelihood estimation of the phase error calculated by phenix.refine is 0.49° less when the IASs are included, suggesting an improvement in map quality (which may indirectly improve the model by aiding in subsequent manual interpretation of electron density features). Final refinement was performed without geometry or ADP weights (unrestrained).

#### Data collection at physiological temperature, refinement, and modeling

We used a low-dose x-ray data collection strategy to acquire diffraction data from macrodomain crystals (C2 crystal form) at human physiological temperature (37°C, 310 K), which is the temperature most relevant to studies of SARS-CoV-2 infection. Using this strategy, we acquired datasets using an x-ray exposure of only 50 kGy, less than 1% of the total dose used at 100 K, which is essential to mitigate the rapid rate of radiation damage at 310 K compared to 100 K. The lower overall x-ray dose resulted in data with a lower overall resolution, extending to 1.5 Å.

Diffraction data from multiple crystals were merged using xia2 (*62*), implementing DIALS (*63*) for indexing and integration, and Aimless (*64*) for scaling and merging. We calculated phases by the method of molecular replacement, using the program Phaser (*58*) and our high-resolution 100K structure as the search model. The model was manually adjusted in Coot to fit the electron density map calculated from molecular replacement, followed by automated refinement of coordinates, atomic displacement parameters, and occupancies using phenix.refine (*60*) with optimization of restraint weights.

#### Fragment data collection, refinement, and modeling

Diffraction data were collected at ALS beamline 8.3.1 and SSRL beamlines 12-1 and 12-2. The data collection strategy is summarized in data file S1. Fragment datasets were indexed, integrated, and scaled using XDS (*57*) run through xia2 (*62*). On the basis of the space group and unit cell dimensions, six crystal forms were present (fig. S2C). For each of the three C2 isoforms with one molecule in the ASU (isoforms A, B, and C), a single, high-resolution dataset was selected to create a representative model for each isoform. Phases were obtained via molecular replacement with Phaser (*58*), using the ultrahigh-resolution C2 coordinates as the search model (PDB 7KR0). Coordinates were refined with iterative rounds of manual model building in Coot and refinement with phenix.refine (*60*). Default refinement parameters were used, except the fact that five refinement macrocycles were carried out per iteration and water molecules were automatically added to peaks in the 2mF_O_-DF_C_ electron density map higher than 3.5 σ. The minimum model-water distance was set to 1.8 Å, and a maximum model-water distance was set to 6 Å. For later rounds of refinement, hydrogens were added to riding positions using phenix.ready_set, and B-factors were refined anisotropically for nonhydrogen and nonwater atoms. Although these datasets were obtained from crystals soaked with fragments, there was no evidence for fragment binding in the mF_O_-DF_C_ difference density maps; therefore, the datasets were deemed acceptable as representative DMSO-only models for each isoform.

For the fragment datasets, molecular replacement was performed with Phaser (*58*) and initial refinement with Refmac (*65*), both run through the DIMPLE pipeline (*66*). The search model used for molecular replacement was selected to match the isoform of the dataset. Waters were included in the initial refinement by changing the HOH records in the PDB file to WWW. After refinement, waters were stripped from models, and electron density maps were analyzed for fragment binding using PanDDA (*39*). Electron density maps from 31 datasets were used to calculate the background electron density map for the isoform A, and 24 datasets were used for isoforms B and C (data file S1). Datasets selected for background map calculation had the highest resolution and lowest *R*_free_ values. After PanDDA was run with default parameters, the threshold used to classify a hit was decreased by adjusting the Z-map analysis settings (contour_level = 2, min_blob_volume = 5, min_blob_z_peak = 2.5). Although there was a substantial increase in false positives, the decreased threshold allowed an additional seven fragments to be identified. Fragments were modeled into PanDDA event maps with Coot, using restraints generated by phenix.elbow from a SMILES (simplified molecular-input line-entry system) string (*67*). Changes in protein conformation and solvation were also modeled. Because PanDDA can identify fragments binding with low occupancies, any changes in protein coordinates will have similar, low occupancies. If unrestrained refinement is performed on these low-occupancy models, then changes supported by PanDDA event maps are often reverted to the ground-state model. In the past, this has been overcome by refining both ground-state (apo) and changed-state (fragment bound) structures simultaneously, with the changed state coordinates restrained. However, these multistate models can be difficult to interpret. As an alternative, we modeled and refined the changed state only. To prevent reversion of the model into ground-state density, coordinate refinement was switched off after fragments were modeled. Hydrogens were added with phenix.ready_set, waters were updated automatically, and B-factors were refined anisotropically for nonhydrogen and nonwater atoms. After one round of refinement, waters added into ground-state electron density were removed. This was achieved by aligning the DMSO-only model to the refined model and removing any water molecules within 2.2 Å of the DMSO-only model. A final round of refinement was performed without updating water molecules.

### P4_3_ crystals at UCSF

#### Protein expression and purification

The C2 sequence in pET22b(+) was converted into the P4_3_ sequence by removal of Glu^170^ and replacement of the N-terminal Asn-Ala-Gly-Glu motif with a methionine. In addition, a Ser-Ser-Gly-Val-Asp-Leu-Gly-Thr linker was introduced between the His_6_ tag and the TEV recognition sequence (data file S1). All cloning steps were performed by polymerase chain reaction (PCR) with overlapping primers and Gibson assembly (*68*). Protein was purified using the same protocol as the C2 protein, except that after SEC, the protein was concentrated to 40 mg/ml before flash-freezing in liquid nitrogen.

#### Crystallization

Initially, crystals were grown by hanging-drop vapor diffusion with a reservoir solution containing 34% PEG-3000 and 100 mM N-cyclohexyl-2-aminoethanesulfonic acid (CHES) (pH 9.5). Screens were performed using pregreased VDX plates (Hampton Research, HR3-142) with 0.5 ml of reservoir solution in each well. Crystallization drops were set up on silicon coverslips (Hampton Research, HR3-233) with 2 μl of Mac1 at 10 mg/ml and 2 μl of reservoir. Crystals grew after 2 to 4 days at 19°C. As with the C2 crystals, microseeding was required to achieve consistent nucleation. Seed stocks were prepared as described previously, except the seed storage buffer used was 35% PEG-3000, 100 mM CHES (pH 9.5), and 2 mM DTT. Crystals for fragment soaking were grown using SWISSCI three-well sitting drop plates with reservoirs containing 30 μl of 28% PEG-3000 and 100 mM CHES (pH 9.5). Crystallization drops were set up with 100 nl of reservoir solution, 100 nl of seed stocks (1:100,000 dilution), and 200 nl of Mac1 at 40 mg/ml. Crystals were grown at 19°C and reached a maximum size after 24 hours.

#### Fragment and ADPr soaking

Fragment soaks were performed using the same protocol as the C2 crystals, with soak times between 2 and 6 hours. ADPr soaks were performed similarly, except that ADPr was prepared in water to 100 mM, and crystals were soaked with 80 nl of ADPr (20 mM final concentration). Crystals were vitrified directly after soaking using a NANUQ cryocooling device (MiTeGen).

#### Fragment data collection, processing, modeling, and refinement

Diffraction data were collected at ALS beamline 8.3.1, SSRL beamline 12-1, and NSLS-II beamline 17-ID-2. The data collection strategy is summarized in data file S1. Fragment datasets were indexed, integrated, and scaled using XDS (*57*) and merged with Aimless (*64*). In addition to the fragment soaks, we collected diffraction data for 40 crystals soaked only with DMSO. To generate a DMSO-only model, a single high-resolution dataset was selected, and phases were obtained by molecular replacement using the 0.77-Å C2 structure as a search model (PDB 7KR0). Refinement and model building were performed as described previously for the C2 crystals. The fragment datasets were prepared for PanDDA analysis using the DIMPLE pipeline (*39*, *66*). Fragments were identified using PanDDA, with the background electron density map generated using 35 DMSO-only datasets. As with the analysis of C2 electron density maps, PanDDA was rerun with a decreased Z-map threshold (contour_level = 2.5, min_blob_volume = 5, min_blob_z_peak = 2.5). This strategy identified an additional 24 fragments. Fragment modeling and refinement were carried out using the same protocol as the experiment with C2 crystals.

### P4_3_ crystals at Oxford/XChem

#### Protein expression and purification

SARS-CoV-2 Nsp3 Mac1 (residues 3 to 169) was cloned into a pNIC28-Bsa4 expression vector, which adds an N-terminal His_6_-tag and a TEV protease recognition site for removal of the tag. For expression of protein used for crystallization, the construct was transformed into the *E. coli* Rosetta strain BL21(DE3)-R3, and cells were grown at 37°C in LB medium (Miller) supplemented with kanamycin (50 μg/ml) and chloramphenicol (35 μg/ml). After reaching an optical density at 600 nm of 0.5 to 0.6, the temperature was lowered to 18°C before induction of protein expression overnight by adding 0.5 mM IPTG. Harvested cells were resuspended in lysis buffer [50 mM Hepes (pH 7.5), 500 mM NaCl, 5% glycerol, 20 mM imidazole, 10 mM βME, and cOmplete EDTA-free protease inhibitors (Roche)] and stored at −20°C until purification. For protein purification, pellets were gently thawed in lukewarm water and lysed by high-pressure homogenization. DNA was digested using Benzonase. Proteins were purified by immobilized metal affinity chromatography (IMAC) using Ni-Sepharose resin (GE Healthcare) and eluted stepwise in binding buffer containing 40 to 500 mM imidazole. A high-salt wash with 1 M NaCl was combined with the first elution step including 40 mM imidazole. Removal of the His_6_ tag was carried out by addition of recombinant TEV protease during overnight dialysis into buffer without imidazole, followed by purification on a second IMAC column, and lastly by SEC (Superdex 75, GE Healthcare) in a buffer consisting of 20 mM Hepes (pH 8.0), 250 mM NaCl, and 2 mM DTT. Macrodomain protein used for HTRF assay was not subjected to TEV cleavage and purified after the IMAC step by SEC in a buffer consisting of 25 mM Hepes (pH 7.4), 300 mM NaCl, 5% glycerol, and 0.5 mM tris(2-carboxyethyl)phosphine. Proteins were characterized by SDS–polyacrylamide gel electrophoresis, then flash-frozen in liquid nitrogen, and stored at −80°C until required.

#### Crystallographic fragment screening

SARS-CoV-2 Nsp3 Mac1 was concentrated to a final concentration of 47 mg/ml and apo crystals were grown in crystallization solution containing 100 mM CHES (pH 9.5) and 30% PEG-3000. Fragments were soaked into crystals as previously described (*23*) by adding dissolved compounds directly to the crystallization drops using an Echo liquid handler (final concentration, 10% DMSO); drops were incubated for approximately 1 to 3 hours before mounting and flash-freezing in liquid nitrogen.

Data were collected at the beamline I04-1 at 100 K and automatically processed with Diamond Light Source’s autoprocessing pipelines using XDS (*57*) and either xia2 (*62*) or DIALS (*63*) with the default settings. Most Mac1 data processed to a resolution of approximately 1.1 Å. Further analysis was performed with XChemExplorer (*24*), electron density maps were generated with DIMPLE (*66*), and ligand-binding events were identified using PanDDA (*39*). Ligands were modeled into PanDDA-calculated event maps using Coot (*59*), restraints were calculated with AceDRG (*69*), and structures were refined with BUSTER (*70*). Coordinates, structure factors, and PanDDA event maps for the structures discussed are deposited in the PDB. Data collection and refinement statistics are summarized in data file S1.

### Molecular docking screens

Docking was performed against the crystal structure of SARS-CoV-2 Nsp3 Mac1 bound to ADPr (PDB 6W02) (*34*). Chain B and all water molecules except for HOH324, HOH344, HOH384, and HOH406 were removed. These water molecules were included in the docking template structure because they were buried within the ADPr-binding site and formed bridging hydrogen bonds between ADPr and the protein. The protein structure in complex with ADPr and the four selected water molecules was capped at N and C termini and prepared for docking following the prepwizard protocol in Maestro (Schrödinger) (*71*). Accordingly, protons were added using Epik, and protonation states were optimized with PropKa at pH 7. Last, the structure was energetically minimized using the OPLS3e force field (*71*). The maximum heavy-atom deviation from the initial structure was 0.3 Å (*71*).

Docking was performed with DOCK3.7 using precalculated scoring grids for rapid evaluation of docked molecules (*72*). AMBER united-atom charges (*73*) were assigned to the minimized protein structure and water molecules. Partial atomic charges of backbone amide hydrogen atoms for residues Ile^23^ and Phe^156^ were increased by 0.2 elementary charge units without changing the net charge of the residues, as described previously (*29*). The low dielectric constant of the protein environment was extended outwards from the protein surface by 1.9 Å using spheres generated by Sphgen. Electrostatic potentials at the ligand-binding pocket were calculated by the numerical solution of the Poisson-Boltzmann equation using QNIFFT (*74*), and scoring grids for van der Waals potentials were generated with CHEMGRID. Ligand desolvation scoring grids were calculated by Solvmap (*75*), and the volume of the low-protein dielectric was extended out 0.4 Å from the protein surface, as described previously (*40*). Because we specifically targeted the adenosine-binding site of the full ADPr-binding pocket, atomic coordinates of adenosine rather than the whole ADPr molecule were used to generate 45 matching spheres, representing favorable positions for placing ligand atoms with docking (*72*).

As ADPr was the only known ligand for Mac1 when we started the docking campaign, the generated scoring grids and matching spheres were judged for their ability to place and score adenosine, adenine, and ribose at the adenosine-binding site of the ligand-binding pocket compared to 250 property-matched decoys, generated following the DUDE-Z method (*76*). Decoys share similar physical properties as the control molecules but are topologically different, hence unlikely to ligate the binding pocket. Furthermore, an “extrema” set (*76*) of approximately 500,000 molecules including anionic, neutral, and cationic compounds with molecular weights ranging from 250 to 350 Da was screened to ensure similar enrichments for monovalent anions and neutral molecules. We note that the lack of experimentally confirmed ligands for the macrodomain did not allow exhaustive control calculations.

Virtual compound libraries were downloaded from ZINC15 (http://zinc15.docking.org) (*37*). From the set of 722,963 in-stock fragments, 696,092 compounds were successfully docked, exploring on average 2355 orientations and 63 conformations per compound in the binding pocket. Roughly 58 billion complexes were sampled in 88 core hours or roughly 10 min on a 500-core cluster. Screening the entire 20 million ZINC15 fragment library resulted in the evaluation of ca. 4.4 trillion complexes within 2342 core hours or 4.7 hours on 500 cores. In that screen, 19,130,798 compounds were scored and sampled in ca. 2145 orientations and 180 conformations each. From the relatively small in-human library, containing 20,726 molecules, 17,362 compounds were scored, and sampling was increased to roughly 16,615 orientations per compound. Eighty-four billion complexes were evaluated in 27 core hours.

Compounds with DOCK scores of <−20 (top 500,000 compounds from the entire fragment screen) were subsequently filtered for those with strained conformations and inspected for their ability to form hydrogen bonds to residues Asp^22^, Ile^23^, Gly^48^, Val^49^, Gly^130^, or Phe^156^. Compounds with unsatisfied hydrogen bond donors or more than three unsatisfied hydrogen bond acceptors were deprioritized. From both fragment screens, 17 in-stock compounds (8 selected from the ZINC15 in-stock library docking screen) were purchased, and 45 make-on-demand fragments were ordered of which 33 were successfully synthesized, both from Enamine. The following compounds were selected from the in-human collection docking screen and purchased from different vendors: pterin (Sigma-Aldrich, P1132), verdiperstat (MedChem Express, HY-17646), kinetin (Cayman Chemical, 20712), irsogladine (Cayman Chemical, 30223), diaveridine (Cayman Chemical, 29427), N^6^-benzyladenine (Cayman Chemical, 21711), PP2 (Cayman Chemical, 13198), temozolomide (Cayman Chemical, 14163), chrysophanol (Cayman Chemical, 19870), and isoxanthopterin (Cayman Chemical, 17564).

### Fragment linking and merging

Fragment mergers and linkers were generated using Fragmenstein (https://github.com/matteoferla/Fragmenstein), a python module that automatically joins fragments or places compounds based on fragments in a way that is as faithful to the positions of the fragments as possible in a conformation that is energy acceptable. For merging, using RDKit (*77*), rings are temporarily collapsed into pseudo-atoms, one-to-one spatial overlapping atoms are identified; pseudo-atoms expanded with appropriate bonds to nearby atoms and various chemical corrections applied. For the constrained energy minimization, PyRosetta is used (*78*). Interactive online summary of mergers was made at https://michelanglo.sgc.ox.ac.uk (*79*).

### Differential scanning fluorimetry

Compounds were dissolved in DMSO to a final concentration of 100 mM and placed in a 384-well Echo source plate (Labcyte, PP0200). Using a Labcyte Echo, each compound was dispensed into a 384-well storage plate (Greiner Bio-One, 781280) in five stock concentrations in twofold serial dilutions (compounds, 6.25 to 100 mM; ADPr, 0.625 to 10 mM) and a final volume of 750 nl in triplicate. Two identical plates were created, with the second plate used to provide protein-free controls for all tested conditions. Echo dispensing instructions were created by an in-house app (https://gestwickilab.shinyapps.io/echo_layout_maker/).

DSF buffer was prepared by adding 10 μl of SYPRO Orange (Thermo Fisher Scientific, S6650) to 10 ml of buffer [50 mM tris-HCl (pH 7.5), 150 mM NaCl, 1 mM EDTA, 1 mM DTT, and 0.01% Triton X-100] for a final dye concentration of 5× (10 μM) SYPRO Orange. A compound plate (see above) was resuspended by the addition of 20 μl of DSF buffer and set aside for 20 min in the dark. Purified Mac1 (P4_3_ construct purified at UCSF) was diluted to 10 μM in DSF buffer, and 2 μl of either protein solution or protein-free buffer was added to each well of a 384-well white PCR plate (Axygen, PCR-384-LC480WNFBC) using an E1 ClipTip P125 electronic pipette. Eight microliters of resuspended compound was transferred to each well of the protein- and buffer-containing PCR plate using an Opentrons OT-2 liquid handling system, yielding the following final conditions: 2 μM Mac1, 5× (10 μM) SYPRO Orange, 3% DMSO, 0.1 to 3 mM fragments, and 0.1 to 1 mM ADPr. The PCR plate was spun briefly in a salad spinner to remove bubbles and sealed with an optically clear film (Applied Biosystems, MicroAmp Optical Adhesive Film, 4311971). In an Analytik Jena qTOWER 384G quantitative PCR instrument, plate was continuously heated from 25° to 94°C at a rate of 1°C/min, and fluorescence was measured at each degree in the TAMRA channel (535/580 nm). Fifty-three of 54 fragments could be tested up to 3 mM without assay interference under these conditions (data files S1 and S2). *T*m_a_ values were calculated online at DSFworld, using fitting model 2 (*80*).

Raw DSF data for the Mac1 construct used in this work were characterized by a major transition at 50.8° ± 0.3°C, with a minor second transition at 67° ± 4°C (Fig. 9, C and D, and data files S1 and S2); results described refer to the major transition. Significance was defined as compounds with analysis of variance (ANOVA) *P* < 0.005 for *T*m_a_ over the tested concentration regime.

### Isothermal titration calorimetry

All ITC titrations were performed on a MicroCal iTC 200 instrument (GE Healthcare). All reactions were performed in 20 mM tris (pH 7.5) and 150 mM NaCl using 300 to 600 μM Mac1 (P4_3_ construct purified at UCSF) at 25°C. Titration of 4 mM ADP-ribose (Sigma-Aldrich, A0752) or 4 to 10 mM fragment contained in the stirring syringe included a single 0.2-μl injection, followed by 18 consecutive injections of 2 μl. Data were analyzed using the MicroCal PEAQ-ITC analysis software v1.1.0.1262 (Malvern). Thermograms were integrated and normalized binding enthalpies fitted to an equilibrium binding isotherm (nonlinear least squares fit) using a single-site binding model.

### HTRF-based peptide displacement assay

Fragment inhibitory activity on Mac1 was assessed by the displacement of an ADPr-conjugated biotin peptide from the His_6_-tagged Mac1 using HTRF with an Eu^3+^-conjugated anti-His_6_ antibody donor and streptavidin-conjugated acceptor. Compounds were dispensed into white ProxiPlate-384 Plus (PerkinElmer) assay plates using an Echo 525 liquid handler (Labcyte). Binding assays were conducted in a final volume of 16 μl with 12.5 nM Mac1, 400 nM peptide ARTK(Bio)QTARK(Aoa-RADP)S [synthesized by Cambridge Peptides (Birmingham, UK)], 1:125 streptavidin-XL665 (Cisbio), and 1:20,000 anti–His_6_-Eu^3+^ cryptate (PerkinElmer) in assay buffer [25 mM Hepes (pH 7.0), 20 mM NaCl, 0.05% bovine serum albumin, and 0.05% Tween 20]. Assay reagents were dispensed into plates using a Multidrop combi (Thermo Fisher Scientific) and incubated at room temperature for 1 hour. Fluorescence was measured using a PHERAstar microplate reader (BMG) using the HTRF module with dual-emission protocol (A = excitation of 320 nm, emission of 665 nm and B = excitation of 320 nm, emission of 620 nm). Raw data were processed to give an HTRF ratio (channel A/B × 10,000), which was used to generate IC_50_ curves by nonlinear regression using GraphPad Prism v8 (GraphPad Software, CA, USA).

## SUPPLEMENTARY MATERIALS

Supplementary material for this article is available at http://advances.sciencemag.org/cgi/content/full/7/16/eabf8711/DC1

## Appendix

View/request a protocol for this paper from *Bio-protocol*.

## References

[1] J. G. M. Rack, D. Perina, I. Ahel, Macrodomains: Structure, function, evolution, and catalytic activities. Annu. Rev. Biochem. 85, 431–454 (2016). 26844395 10.1146/annurev-biochem-060815-014935

[2] C. Li, Y. Debing, G. Jankevicius, J. Neyts, I. Ahel, B. Coutard, B. Canard, Viral macro domains reverse protein ADP-ribosylation. J. Virol. 90, 8478–8486 (2016). 27440879 10.1128/JVI.00705-16 PMC5021415

[3] A. R. Fehr, S. A. Singh, C. M. Kerr, S. Mukai, H. Higashi, M. Aikawa, The impact of PARPs and ADP-ribosylation on inflammation and host-pathogen interactions. Genes Dev. 34, 341–359 (2020). 32029454 10.1101/gad.334425.119 PMC7050484

[4] Y. M. O. Alhammad, A. R. Fehr, The viral macrodomain counters host antiviral ADP-ribosylation. Viruses. 12, 384 (2020). 10.3390/v12040384 PMC7232374 32244383

[5] A. R. Fehr, R. Channappanavar, G. Jankevicius, C. Fett, J. Zhao, J. Athmer, D. K. Meyerholz, I. Ahel, S. Perlman, The conserved coronavirus macrodomain promotes virulence and suppresses the innate immune response during severe acute respiratory syndrome coronavirus infection. MBio. 7, e01721-16 (2016). 27965448 10.1128/mBio.01721-16 PMC5156301

[6] A. R. Fehr, J. Athmer, R. Channappanavar, J. M. Phillips, D. K. Meyerholz, S. Perlman, The nsp3 macrodomain promotes virulence in mice with coronavirus-induced encephalitis. J. Virol. 89, 1523–1536 (2015). 25428866 10.1128/JVI.02596-14 PMC4300739

[7] M. E. Grunewald, Y. Chen, C. Kuny, T. Maejima, R. Lease, D. Ferraris, M. Aikawa, C. S. Sullivan, S. Perlman, A. R. Fehr, The coronavirus macrodomain is required to prevent PARP-mediated inhibition of virus replication and enhancement of IFN expression. PLoS Pathog. 15, e1007756 (2019). 31095648 10.1371/journal.ppat.1007756 PMC6521996

[8] L. Palazzo, P. Mikolčević, A. Mikoč, I. Ahel, ADP-ribosylation signalling and human disease. Open Biol. 9, 190041 (2019). 30991935 10.1098/rsob.190041 PMC6501648

[9] G. Caprara, E. Prosperini, V. Piccolo, G. Sigismondo, A. Melacarne, A. Cuomo, M. Boothby, M. Rescigno, T. Bonaldi, G. Natoli, PARP14 controls the nuclear accumulation of a subset of type I IFN-inducible proteins. J. Immunol. 200, 2439–2454 (2018). 29500242 10.4049/jimmunol.1701117

[10] A. R. Fehr, G. Jankevicius, I. Ahel, S. Perlman, Viral macrodomains: Unique mediators of viral replication and pathogenesis. Trends Microbiol. 26, 598–610 (2018). 29268982 10.1016/j.tim.2017.11.011 PMC6003825

[11] J. Cui, F. Li, Z.-L. Shi, Origin and evolution of pathogenic coronaviruses. Nat. Rev. Microbiol. 17, 181–192 (2019). 30531947 10.1038/s41579-018-0118-9 PMC7097006

[12] J. G. M. Rack, V. Zorzini, Z. Zhu, M. Schuller, D. Ahel, I. Ahel, Viral macrodomains: A structural and evolutionary assessment of the pharmacological potential. Open Biol. 10, 200237 (2020). 33202171 10.1098/rsob.200237 PMC7729036

[13] K. K. Eriksson, L. Cervantes-Barragán, B. Ludewig, V. Thiel, Mouse hepatitis virus liver pathology is dependent on ADP-ribose-1″-phosphatase, a viral function conserved in the alpha-like supergroup. JVI. 82, 12325–12334 (2008). 10.1128/JVI.02082-08 PMC2593347 18922871

[14] A. Putics, W. Filipowicz, J. Hall, A. E. Gorbalenya, J. Ziebuhr, ADP-ribose-1-monophosphatase: A conserved coronavirus enzyme that is dispensable for viral replication in tissue culture. J. Virol. 79, 12721–12731 (2005). 16188975 10.1128/JVI.79.20.12721-12731.2005 PMC1235854

[15] D. I. James, K. M. Smith, A. M. Jordan, E. E. Fairweather, L. A. Griffiths, N. S. Hamilton, J. R. Hitchin, C. P. Hutton, S. Jones, P. Kelly, A. E. McGonagle, H. Small, A. I. J. Stowell, J. Tucker, I. D. Waddell, B. Waszkowycz, D. J. Ogilvie, First-in-class chemical probes against poly(ADP-ribose) glycohydrolase (PARG) inhibit DNA repair with differential pharmacology to olaparib. ACS Chem. Biol. 11, 3179–3190 (2016). 27689388 10.1021/acschembio.6b00609

[16] A. I. J. Stowell, D. I. James, I. D. Waddell, N. Bennett, C. Truman, I. M. Hardern, D. J. Ogilvie, A high-throughput screening-compatible homogeneous time-resolved fluorescence assay measuring the glycohydrolase activity of human poly(ADP-ribose) glycohydrolase. Anal. Biochem. 503, 58–64 (2016). 27036617 10.1016/j.ab.2016.03.016

[17] M. Schuller, K. Riedel, I. Gibbs-Seymour, K. Uth, C. Sieg, A. P. Gehring, I. Ahel, F. Bracher, B. M. Kessler, J. M. Elkins, S. Knapp, Discovery of a selective allosteric inhibitor targeting macrodomain 2 of polyadenosine-diphosphate-ribose polymerase 14. ACS Chem. Biol. 12, 2866–2874 (2017). 28991428 10.1021/acschembio.7b00445 PMC6089342

[18] R. S. Virdi, R. V. Bavisotto, N. C. Hopper, N. Vuksanovic, T. R. Melkonian, N. R. Silvaggi, D. N. Frick, Discovery of drug-like ligands for the Mac1 domain of SARS-CoV-2 Nsp3. SLAS Discovery. 25, 1162–1170 (2020). 32981460 10.1177/2472555220960428 PMC7684785

[19] M. M. Hann, A. R. Leach, G. Harper, Molecular complexity and its impact on the probability of finding leads for drug discovery. J. Chem. Inf. Comput. Sci. 41, 856–864 (2001). 11410068 10.1021/ci000403i

[20] C. W. Murray, D. C. Rees, The rise of fragment-based drug discovery. Nat. Chem. 1, 187–192 (2009). 21378847 10.1038/nchem.217

[21] T. Krojer, J. S. Fraser, F. von Delft, Discovery of allosteric binding sites by crystallographic fragment screening. Curr. Opin. Struct. Biol. 65, 209–216 (2020). 33171388 10.1016/j.sbi.2020.08.004 PMC10979522

[22] D. A. Erlanson, S. W. Fesik, R. E. Hubbard, W. Jahnke, H. Jhoti, Twenty years on: The impact of fragments on drug discovery. Nat. Rev. Drug Discov. 15, 605–619 (2016). 27417849 10.1038/nrd.2016.109

[23] P. M. Collins, J. T. Ng, R. Talon, K. Nekrosiute, T. Krojer, A. Douangamath, J. Brandao-Neto, N. Wright, N. M. Pearce, F. von Delft, Gentle, fast and effective crystal soaking by acoustic dispensing. Acta Crystallogr D Struct Biol. 73, 246–255 (2017). 28291760 10.1107/S205979831700331X PMC5349437

[24] T. Krojer, R. Talon, N. Pearce, P. Collins, A. Douangamath, J. Brandao-Neto, A. Dias, B. Marsden, F. von Delft, The XChemExplorer graphical workflow tool for routine or large-scale protein-ligand structure determination. Acta Crystallogr D Struct Biol. 73, 267–278 (2017). 28291762 10.1107/S2059798316020234 PMC5349439

[25] N. D. Wright, P. Collins, R. Talon, E. Nelson, L. Koekemoer, M. Ye, R. Nowak, J. Newman, J. T. Ng, N. Mitrovich, H. Wiggers, F. von Delft, The low-cost, semi-automated shifter microscope stage transforms speed and robustness of manual protein crystal harvesting. bioRxiv 2019.12.20.875674 [**Preprint**]. 20 December 2019. 10.1101/2019.12.20.875674.

[26] A. Douangamath, D. Fearon, P. Gehrtz, T. Krojer, P. Lukacik, C. D. Owen, E. Resnick, C. Strain-Damerell, A. Aimon, P. Ábrányi-Balogh, J. Brandaõ-Neto, A. Carbery, G. Davison, A. Dias, T. D. Downes, L. Dunnett, M. Fairhead, J. D. Firth, S. P. Jones, A. Keely, G. M. Keserü, H. F. Klein, M. P. Martin, M. E. M. Noble, P. O’Brien, A. Powell, R. Reddi, R. Skyner, M. Snee, M. J. Waring, C. Wild, N. London, F. von Delft, M. A. Walsh, Crystallographic and electrophilic fragment screening of the SARS-CoV-2 main protease. Nat. Commun. 11, 5047 (2020). 33028810 10.1038/s41467-020-18709-w PMC7542442

[27] R. J. Hall, P. N. Mortenson, C. W. Murray, Efficient exploration of chemical space by fragment-based screening. Prog. Biophys. Mol. Biol. 116, 82–91 (2014). 25268064 10.1016/j.pbiomolbio.2014.09.007

[28] A. Manglik, H. Lin, D. K. Aryal, J. D. McCorvy, D. Dengler, G. Corder, A. Levit, R. C. Kling, V. Bernat, H. Hübner, X.-P. Huang, M. F. Sassano, P. M. Giguère, S. Löber, D. Duan, G. Scherrer, B. K. Kobilka, P. Gmeiner, B. L. Roth, B. K. Shoichet, Structure-based discovery of opioid analgesics with reduced side effects. Nature. 537, 185–190 (2016). 27533032 10.1038/nature19112 PMC5161585

[29] J. Lyu, S. Wang, T. E. Balius, I. Singh, A. Levit, Y. S. Moroz, M. J. O’Meara, T. Che, E. Algaa, K. Tolmachova, A. A. Tolmachev, B. K. Shoichet, B. L. Roth, J. J. Irwin, Ultra-large library docking for discovering new chemotypes. Nature. 566, 224–229 (2019). 30728502 10.1038/s41586-019-0917-9 PMC6383769

[30] R. M. Stein, H. J. Kang, J. D. McCorvy, G. C. Glatfelter, A. J. Jones, T. Che, S. Slocum, X.-P. Huang, O. Savych, Y. S. Moroz, B. Stauch, L. C. Johansson, V. Cherezov, T. Kenakin, J. J. Irwin, B. K. Shoichet, B. L. Roth, M. L. Dubocovich, Virtual discovery of melatonin receptor ligands to modulate circadian rhythms. Nature. 579, 609–614 (2020). 32040955 10.1038/s41586-020-2027-0 PMC7134359

[31] Y. Bian, X.-Q. S. Xie, Computational fragment-based drug design: Current trends, strategies, and applications. AAPS J. 20, 59 (2018). 29633051 10.1208/s12248-018-0216-7 PMC6618289

[32] Y. Chen, B. K. Shoichet, Molecular docking and ligand specificity in fragment-based inhibitor discovery. Nat. Chem. Biol. 5, 358–364 (2009). 19305397 10.1038/nchembio.155 PMC4006998

[33] D. G. Teotico, K. Babaoglu, G. J. Rocklin, R. S. Ferreira, A. M. Giannetti, B. K. Shoichet, Docking for fragment inhibitors of AmpC beta-lactamase. Proc. Natl. Acad. Sci. U. S. A. 106, 7455–7460 (2009). 19416920 10.1073/pnas.0813029106 PMC2671983

[34] K. Michalska, Y. Kim, R. Jedrzejczak, N. I. Maltseva, L. Stols, M. Endres, A. Joachimiak, Crystal structures of SARS-CoV-2 ADP-ribose phosphatase: From the apo form to ligand complexes. IUCrJ. 7, 814–824 (2020). 32939273 10.1107/S2052252520009653 PMC7467174

[35] T. S. Walter, C. Meier, R. Assenberg, K.-F. Au, J. Ren, A. Verma, J. E. Nettleship, R. J. Owens, D. I. Stuart, J. M. Grimes, Lysine methylation as a routine rescue strategy for protein crystallization. Structure. 14, 1617–1622 (2006). 17098187 10.1016/j.str.2006.09.005 PMC7126202

[36] O. B. Cox, T. Krojer, P. Collins, O. Monteiro, R. Talon, A. Bradley, O. Fedorov, J. Amin, B. D. Marsden, J. Spencer, F. von Delft, P. E. Brennan, A poised fragment library enables rapid synthetic expansion yielding the first reported inhibitors of PHIP(2), an atypical bromodomain. Chem. Sci. 7, 2322–2330 (2016). 29910922 10.1039/c5sc03115j PMC5977933

[37] T. Sterling, J. J. Irwin, ZINC 15—Ligand discovery for everyone. J. Chem. Inf. Model. 55, 2324–2337 (2015). 26479676 10.1021/acs.jcim.5b00559 PMC4658288

[38] G. W. Bemis, M. A. Murcko, The properties of known drugs. 1. Molecular frameworks. J. Med. Chem. 39, 2887–2893 (1996). 8709122 10.1021/jm9602928

[39] N. M. Pearce, T. Krojer, A. R. Bradley, P. Collins, R. P. Nowak, R. Talon, B. D. Marsden, S. Kelm, J. Shi, C. M. Deane, F. von Delft, A multi-crystal method for extracting obscured crystallographic states from conventionally uninterpretable electron density. Nat. Commun. 8, 15123 (2017). 28436492 10.1038/ncomms15123 PMC5413968

[40] M. M. Mysinger, D. R. Weiss, J. J. Ziarek, S. Gravel, A. K. Doak, J. Karpiak, N. Heveker, B. K. Shoichet, B. F. Volkman, Structure-based ligand discovery for the protein-protein interface of chemokine receptor CXCR4. Proc. Natl. Acad. Sci. U. S. A. 109, 5517–5522 (2012). 22431600 10.1073/pnas.1120431109 PMC3325704

[41] W. J. Allen, R. C. Rizzo, Implementation of the Hungarian algorithm to account for ligand symmetry and similarity in structure-based design. J. Chem. Inf. Model. 54, 518–529 (2014). 24410429 10.1021/ci400534h PMC3958141

[42] D. Ramírez, J. Caballero, Is it reliable to take the molecular docking top scoring position as the best solution without considering available structural data? Molecules. 23, 1038 (2018). 10.3390/molecules23051038 PMC6102569 29710787

[43] L. Xing, J. Klug-Mcleod, B. Rai, E. A. Lunney, Kinase hinge binding scaffolds and their hydrogen bond patterns. Bioorg. Med. Chem. 23, 6520–6527 (2015). 26358279 10.1016/j.bmc.2015.08.006

[44] A. Narunsky, A. Kessel, R. Solan, V. Alva, R. Kolodny, N. Ben-Tal, On the evolution of protein-adenine binding. Proc. Natl. Acad. Sci. U. S. A. 117, 4701–4709 (2020). 32079721 10.1073/pnas.1911349117 PMC7060716

[45] J. S. Fraser, H. van den Bedem, A. J. Samelson, P. T. Lang, J. M. Holton, N. Echols, T. Alber, Accessing protein conformational ensembles using room-temperature x-ray crystallography. Proc. Natl. Acad. Sci. U. S. A. 108, 16247–16252 (2011). 21918110 10.1073/pnas.1111325108 PMC3182744

[46] D. A. Keedy, Z. B. Hill, J. T. Biel, E. Kang, T. J. Rettenmaier, J. Brandão-Neto, N. M. Pearce, F. von Delft, J. A. Wells, J. S. Fraser, An expanded allosteric network in PTP1B by multitemperature crystallography, fragment screening, and covalent tethering. Elife 7, e36307 (2018). 29877794 10.7554/eLife.36307 PMC6039181

[47] A. S. Bayden, D. T. Moustakas, D. Joseph-McCarthy, M. L. Lamb, evaluating free energies of binding and conservation of crystallographic waters using SZMAP. J. Chem. Inf. Model. 55, 1552–1565 (2015). 26176600 10.1021/ci500746d

[48] D. Cappel, W. Sherman, T. Beuming, Calculating water thermodynamics in the binding site of proteins - Applications of watermap to drug discovery. Curr. Top. Med. Chem. 17, 2586–2598 (2017). 28413953 10.2174/1568026617666170414141452

[49] J. J. Irwin, K. G. Tang, J. Young, C. Dandarchuluun, B. R. Wong, M. Khurelbaatar, Y. S. Moroz, J. Mayfield, R. A. Sayle, ZINC20-A free ultralarge-scale chemical database for ligand discovery. J. Chem. Inf. Model. 60, 6065–6073 (2020). 33118813 10.1021/acs.jcim.0c00675 PMC8284596

[50] G. Jankevicius, M. Hassler, B. Golia, V. Rybin, M. Zacharias, G. Timinszky, A. G. Ladurner, A family of macrodomain proteins reverses cellular mono-ADP-ribosylation. Nat. Struct. Mol. Biol. 20, 508–514 (2013). 23474712 10.1038/nsmb.2523 PMC7097781

[51] D. Munnur, E. Bartlett, P. Mikolčević, I. T. Kirby, J. G. M. Rack, A. Mikoč, M. S. Cohen, I. Ahel, Reversible ADP-ribosylation of RNA. Nucleic Acids Res. 47, 5658–5669 (2019). 31216043 10.1093/nar/gkz305 PMC6582358

[52] B. Lamoree, R. E. Hubbard, Current perspectives in fragment-based lead discovery (FBLD). Essays Biochem. 61, 453–464 (2017). 29118093 10.1042/EBC20170028 PMC5869234

[53] M. O’Reilly, A. Cleasby, T. G. Davies, R. J. Hall, R. F. Ludlow, C. W. Murray, D. Tisi, H. Jhoti, Crystallographic screening using ultra-low-molecular-weight ligands to guide drug design. Drug Discov. Today. 24, 1081–1086 (2019). 30878562 10.1016/j.drudis.2019.03.009

[54] D. J. Wood, J. D. Lopez-Fernandez, L. E. Knight, I. Al-Khawaldeh, C. Gai, S. Lin, M. P. Martin, D. C. Miller, C. Cano, J. A. Endicott, I. R. Hardcastle, M. E. M. Noble, M. J. Waring, FragLites–Minimal, halogenated fragments displaying pharmacophore doublets. An efficient approach to druggability assessment and hit generation. J. Med. Chem. 62, 3741–3752 (2019). 30860382 10.1021/acs.jmedchem.9b00304

[55] T. D. Downes, S. P. Jones, H. F. Klein, M. C. Wheldon, M. Atobe, P. S. Bond, J. D. Firth, N. S. Chan, L. Waddelove, R. E. Hubbard, D. C. Blakemore, C. De Fusco, S. D. Roughley, L. R. Vidler, M. A. Whatton, A. J.-A. Woolford, G. L. Wrigley, P. O’Brien, Design and synthesis of 56 shape-diverse 3D fragments. Chemistry 26, 8969–8975 (2020). 32315100 10.1002/chem.202001123 PMC7496344

[56] J. E. Tropea, S. Cherry, D. S. Waugh, Expression and purification of soluble His6-Tagged TEV protease. Methods Mol. Biol. 498, 297–307 (2009). 18988033 10.1007/978-1-59745-196-3_19

[57] W. Kabsch, XDS. Acta Crystallogr. D Biol. Crystallogr. 66, 125–132 (2010). 20124692 10.1107/S0907444909047337 PMC2815665

[58] A. J. McCoy, R. W. Grosse-Kunstleve, P. D. Adams, M. D. Winn, L. C. Storoni, R. J. Read, Phasercrystallographic software. J. Appl. Crystallogr. 40, 658–674 (2007). 19461840 10.1107/S0021889807021206 PMC2483472

[59] P. Emsley, B. Lohkamp, W. G. Scott, K. Cowtan, Features and development of Coot. Acta Crystallogr. D Biol. Crystallogr. 66, 486–501 (2010). 20383002 10.1107/S0907444910007493 PMC2852313

[60] P. V. Afonine, R. W. Grosse-Kunstleve, N. Echols, J. J. Headd, N. W. Moriarty, M. Mustyakimov, T. C. Terwilliger, A. Urzhumtsev, P. H. Zwart, P. D. Adams, Towards automated crystallographic structure refinement with phenix.refine. Acta Crystallogr. D Biol. Crystallogr. 68, 352–367 (2012). 22505256 10.1107/S0907444912001308 PMC3322595

[61] P. V. Afonine, R. W. Grosse-Kunstleve, P. D. Adams, V. Y. Lunin, A. Urzhumtsev, On macromolecular refinement at subatomic resolution with interatomic scatterers. Acta Crystallogr. D Biol. Crystallogr. 63, 1194–1197 (2007). 18007035 10.1107/S0907444907046148 PMC2808317

[62] G. Winter, *xia2*: An expert system for macromolecular crystallography data reduction. J. Appl. Crystallogr. 43, 186–190 (2009).

[63] G. Winter, D. G. Waterman, J. M. Parkhurst, A. S. Brewster, R. J. Gildea, M. Gerstel, L. Fuentes-Montero, M. Vollmar, T. Michels-Clark, I. D. Young, N. K. Sauter, G. Evans, DIALS: Implementation and evaluation of a new integration package. Acta Crystallogr D Struct Biol. 74, 85–97 (2018). 29533234 10.1107/S2059798317017235 PMC5947772

[64] M. D. Winn, C. C. Ballard, K. D. Cowtan, E. J. Dodson, P. Emsley, P. R. Evans, R. M. Keegan, E. B. Krissinel, A. G. W. Leslie, A. McCoy, S. J. McNicholas, G. N. Murshudov, N. S. Pannu, E. A. Potterton, H. R. Powell, R. J. Read, A. Vagin, K. S. Wilson, Overview of the CCP4 suite and current developments. Acta Crystallogr. D Biol. Crystallogr. 67, 235–242 (2011). 21460441 10.1107/S0907444910045749 PMC3069738

[65] G. N. Murshudov, A. A. Vagin, E. J. Dodson, Refinement of macromolecular structures by the maximum-likelihood method. Acta Crystallogr. D Biol. Crystallogr. 53, 240–255 (1997). 15299926 10.1107/S0907444996012255

[66] M. Wojdyr, R. Keegan, G. Winter, A. Ashton, DIMPLE—A pipeline for the rapid generation of difference maps from protein crystals with putatively bound ligands. Acta Crystallogr. Sec. A Found. Crystallogr. 69, s299–s299 (2013).

[67] N. W. Moriarty, R. W. Grosse-Kunstleve, P. D. Adams, electronic Ligand Builder and Optimization Workbench (eLBOW): A tool for ligand coordinate and restraint generation. Acta Crystallogr. D Biol. Crystallogr. 65, 1074–1080 (2009). 19770504 10.1107/S0907444909029436 PMC2748967

[68] D. G. Gibson, L. Young, R.-Y. Chuang, J. C. Venter, C. A. Hutchison III, H. O. Smith, Enzymatic assembly of DNA molecules up to several hundred kilobases. Nat. Methods. 6, 343–345 (2009). 19363495 10.1038/nmeth.1318

[69] F. Long, R. A. Nicholls, P. Emsley, S. Graǽulis, A. Merkys, A. Vaitkus, G. N. Murshudov, AceDRG: A stereochemical description generator for ligands. Acta Crystallogr D Struct Biol. 73, 112–122 (2017). 28177307 10.1107/S2059798317000067 PMC5297914

[70] G. Bricogne, Direct phase determination by entropy maximization and likelihood ranking: Status report and perspectives. Acta Crystallogr. D Biol. Crystallogr. 49, 37–60 (1993). 15299544 10.1107/S0907444992010400

[71] G. M. Sastry, M. Adzhigirey, T. Day, R. Annabhimoju, W. Sherman, Protein and ligand preparation: Parameters, protocols, and influence on virtual screening enrichments. J. Comput. Aided Mol. Des. 27, 221–234 (2013). 23579614 10.1007/s10822-013-9644-8

[72] R. G. Coleman, M. Carchia, T. Sterling, J. J. Irwin, B. K. Shoichet, Ligand pose and orientational sampling in molecular docking. PLOS ONE 8, e75992 (2013). 24098414 10.1371/journal.pone.0075992 PMC3787967

[73] S. J. Weiner, P. A. Kollman, D. A. Case, U. C. Singh, C. Ghio, G. Alagona, S. Profeta, P. Weiner, A new force field for molecular mechanical simulation of nucleic acids and proteins. J. Am. Chem. Soc. 106, 765–784 (1984).

[74] K. Gallagher, K. Sharp, Electrostatic contributions to heat capacity changes of DNA-ligand binding. Biophys. J. 75, 769–776 (1998). 9675178 10.1016/S0006-3495(98)77566-6 PMC1299751

[75] M. M. Mysinger, B. K. Shoichet, Rapid context-dependent ligand desolvation in molecular docking. J. Chem. Inf. Model. 50, 1561–1573 (2010). 20735049 10.1021/ci100214a

[76] R. M. Stein, Y. Yang, T. E. Balius, M. J. O’Meara, J. Lyu, J. Young, K. Tang, B. K. Shoichet, J. J. Irwin, Property-unmatched decoys in docking benchmarks. J. Chem. Inf. Model. 61, 699–714 (2021). 33494610 10.1021/acs.jcim.0c00598 PMC7913603

[77] P. Tosco, N. Stiefl, G. Landrum, Bringing the MMFF force field to the RDKit: Implementation and validation. J. Cheminform. 6, 37 (2014).

[78] S. Chaudhury, S. Lyskov, J. J. Gray, PyRosetta: A script-based interface for implementing molecular modeling algorithms using Rosetta. Bioinformatics 26, 689–691 (2010). 20061306 10.1093/bioinformatics/btq007 PMC2828115

[79] M. P. Ferla, A. T. Pagnamenta, D. Damerell, J. C. Taylor, B. D. Marsden, MichelaNglo: Sculpting protein views on web pages without coding. Bioinformatics 36, 3268–3270 (2020). 32061125 10.1093/bioinformatics/btaa104 PMC7214029

[80] T. Wu, J. Yu, Z. Gale-Day, A. Woo, A. Suresh, M. Hornsby, J. E. Gestwicki, Three essential resources to improve differential scanning fluorimetry (DSF) experiments. Cold Spring Harbor Laboratory, 2020.03.22.002543 (2020).

[81] A. Morin, B. Eisenbraun, J. Key, P. C. Sanschagrin, M. A. Timony, M. Ottaviano, P. Sliz, Cutting edge: Collaboration gets the most out of software. Elife 2, e01456 (2013). 24040512 10.7554/eLife.01456 PMC3771563

[82] M. N. S. Rad, S. Behrouz, E. Zarenezhad, N. Kaviani, Highly efficient protocol for one-pot N-alkylation of nucleobases using alcohols in bmim[Br]: A rapid route to access acyclic nucleosides. J. Iran. Chem. Soc. 12, 1603–1612 (2015).

